# Molecular characterization of Sm*tdc-1* and Sm*ddc-1* discloses roles as male-competence factors for the sexual maturation of *Schistosoma mansoni* females

**DOI:** 10.3389/fcimb.2023.1173557

**Published:** 2023-05-26

**Authors:** Xuesong Li, Oliver Weth, Simone Haeberlein, Christoph G. Grevelding

**Affiliations:** Institute for Parasitology, Biomedical Research Center Seltersberg (BFS), Justus Liebig University, Giessen, Germany

**Keywords:** *Schistosoma mansoni*, male-female interaction, gonad differentiation, L-tyrosine decarboxylase, aromatic-L-amino-acid decarboxylase, neuronal signalling, biogenic amine

## Abstract

**Introduction:**

Schistosomes are the only mammalian flatworms that have evolved separate sexes. A key question of schistosome research is the male-dependent sexual maturation of the female since a constant pairing contact with a male is required for the onset of gonad development in the female. Although this phenomenon is long known, only recently a first peptide-based pheromone of males was identified that contributes to the control of female sexual development. Beyond this, our understanding of the molecular principles inducing the substantial developmental changes in a paired female is still rudimentary.

**Objectives:**

Previous transcriptomic studies have consistently pointed to neuronal genes being differentially expressed and upregulated in paired males. These genes included Smp_135230 and Smp_171580, both annotated as aromatic-L-amino-acid decarboxylases (DOPA decarboxylases). Here, we characterized both genes and investigated their roles in male–female interaction of *S. mansoni*.

**Methodologies/findings:**

Sequence analyses indicated that Smp_135230 represents an L-tyrosine decarboxylase (Sm*tdc-1*), whereas Smp_171580 represents a DOPA decarboxylase (Sm*ddc-1*). By qRT-PCR, we confirmed the male-specific and pairing-dependent expression of both genes with a significant bias toward paired males. RNA-interference experiments showed a strong influence of each gene on gonad differentiation in paired females, which was enhanced by double knockdown. Accordingly, egg production was significantly reduced. By confocal laser scanning microscopy, a failure of oocyte maturation was found in paired knockdown females. Whole-mount *in situ* hybridization patterns exhibited the tissue-specific occurrence of both genes in particular cells at the ventral surface of the male, the gynecophoral canal, which represents the physical interface of both genders. These cells probably belong to the predicted neuronal cluster 2 of *S. mansoni.*

**Conclusion:**

Our results suggest that Sm*tdc-1* and Sm*ddc-2* are male-competence factors that are expressed in neuronal cells at the contact zone between the genders as a response of pairing to subsequently control processes of female sexual maturation.

## 1 Introduction

Schistosome parasites exhibit a remarkable sexual biology. The full development of the ovary and vitellarium, both parts of the female gonad, requires a close and continuous pairing contact of both partners (Popiel and Basch, 1984a; Hollander and Erasmus, 1985; Kunz, 2001). To achieve this, the male encloses the female in its ventral groove, the gynecophoral canal. This way, a stable pairing contact is established that can persist for years. At the same time, constant pairing ensures the onset and continuation of egg production (Basch, 1991; Grevelding, 2004). The latter has fatal consequences for potential hosts of schistosomes, human, and animals and leads to schistosomiasis, a neglected tropical disease (NTD) of worldwide importance. While part of the eggs reaches the environment *via* feces or urine (depending on the schistosome species), the remaining eggs are trapped in organs like liver and spleen where they cause inflammatory processes and fibrosis (Olveda et al., 2014; McManus et al., 2018; World Health Organization, 2021).

In the pregenomic era of schistosome research, it was shown that the male upon pairing governs the expression of female specifically expressed genes with gonadal functions such as egg-shell precursor genes (LoVerde and Chen, 1991; Grevelding et al., 1997; Kunz, 2001). Compared with intensive research activities on females (Hoffmann, 2004; LoVerde et al., 2009; Beckmann et al., 2010), pairing-dependent processes in males have been less investigated. In contrast to females, males have fully developed gonads and differentiated sperm independent of pairing (Neves et al., 2005; Beckmann et al., 2010). Studies in the past excluded sperm as a female maturation-inducing factor since anorchid males were still able to pair with females and to induce their sexual maturation, including egg production (Armstrong, 1965; Michaels, 1969). Alternatively, specific molecules like hormonal factors or peptides were hypothesized to be delivered from the male to the female directing sexual maturation (Armstrong, 1965; Michaels, 1969; Ruppel and Cioli, 1977; Shaw et al., 1977; Atkinson and Atkinson, 1980; Popiel and Basch, 1984a; Popiel and Basch, 1984b; Basch and Nicolas, 1989). Indeed, a recent study uncovered a male-derived peptide-based pheromone, β-alanyl-tryptamine, which is produced by a non-ribosomal peptide synthetase, as one factor involved in controlling female sexual maturation (Chen et al., 2022). Until today, however, it is unclear whether further genes/factors may be involved in the pairing-dependent processes leading to female sexual maturation.

During the last 15 years, *omic* studies based on microarrays, serial analysis of gene expression (SAGE), and RNA-seq have opened up new vistas for addressing unanswered questions of schistosome biology (Hu et al., 2003; Verjovski-Almeida et al., 2003; Fitzpatrick and Hoffmann, 2006; Berriman et al., 2009; Schistosoma japonicum Genome Sequencing and Functional Analysis Consortium, 2009; Protasio et al., 2012; Young et al., 2012; Anderson et al., 2015; Cai et al., 2016; Wang et al., 2017). This included the role of the male in studies comparing pairing-experienced males (bisex males, bM) with pairing-unexperienced ones (single-sex males, sM). In our lab, previous studies combining SuperSAGE and microarray analyses detected 29 genes by both methods as being significantly differentially transcribed between bM and sM of *S. mansoni* (Leutner et al., 2013). Among these were genes involved in neuronal processes, which we also found in subsequent RNA-seq studies including bM and sM (Lu et al., 2016; Lu et al., 2017). Two of these genes were annotated as DOPA decarboxylases (DDC or AADC; E.C.4.1.1.28), Smp_135230 and Smp_171580, enzymes that catalyze decarboxylation reactions of neurotransmitters and neuromodulators (De Luca et al., 2003; Bertoldi, 2014). For both genes, RNA-seq showed significantly higher transcript levels in bM compared with sM, and no transcripts in pairing-experienced (bisex females, bF) or pairing-unexperienced (single-sex females, sF) females (Lu et al., 2016; Lu et al., 2018). Results of a first qRT-PCR analysis with RNA from males following pairing, separation, and re-pairing *in vitro* supported the RNA-seq data and demonstrated the influence of pairing on the upregulation of Sm*ddc*-1 in bM (Haeberlein et al., 2019).

These transcriptomics results led to the hypothesis of male-competence factors, which were defined as gender-specifically and/or preferentially as well as pairing-dependently regulated genes of males with roles in the induction and/or maintenance of female sexual maturation (Lu et al., 2019). To this end, our new study addressed questions about the phylogenetic relationship, pairing-dependent regulation in males, tissue-specific localization, and functional roles of Smp_135230 and Smp_171580. Based on improved *in vitro*-culture conditions for re-pairing experiments of *S. mansoni in vitro*, we performed experiments to functionally characterize these genes and provide conclusive evidence that they are part of the molecular communication during male–female interaction. Both genes are male-specifically/preferentially transcribed and in a pairing-dependent manner, they locate in neuronal cells of the male’s ventral surface that faces the female during pairing, and they control ovary differentiation as part of female sexual maturation.

## 2 Materials and methods

### 2.1 *S. mansoni* maintenance and ethical statement

Freshwater snails (*Biomphalaria glabrata*) served as the intermediate hosts for the Liberian strain of *S. mansoni* maintained in our lab (Grevelding et al., 1997). As final hosts, we used Syrian hamsters (*Mesocricetus auratus*) in accordance with the European Convention for the Protection of Vertebrate Animals used for Experimental and Other Scientific Purposes (ETS No 123; revised Appendix A). The experiments were approved by the Regional Council (Regierungspraesidium) Giessen (V54-19 c 20/15 h 02 GI 18/10 Nr. A 26/2018). Hamsters (Janvier, France) in the age of 8 weeks were infected by the paddling method (Dettman et al., 1989) using up to 1,750 cercariae per hamster. We infected hamsters with cercariae of both genders (bisex, bs) and collected worms by hepatoportal perfusion 46 days (d) postinfection (p.i.). In case of infection with single-sex (ss) worms, snails were infected by a single miracidium each (mono-miracidial infection) to obtain clonal cercariae for infection. In this case, perfusion occurred 67 d p.i. (Grevelding et al., 1997).

### 2.2 *In vitro* culture and pairing experiments with *S. mansoni*

For regular maintenance *in vitro*, we cultured worms in M199 medium (Sigma-Aldrich, Germany) supplemented with 10% newborn calf serum (Sigma), 1% 1 M HEPES, and 1% ABAM solution (10,000 units/ml penicillin, 10 mg/ml streptomycin, and 25 mg/ml amphotericin B; Sigma-Aldrich, Germany) at 37°C in a 5% CO_2_ atmosphere (Grevelding et al., 1997). For pairing and re-pairing experiments, we also made use of the recently developed ABC169 *in vitro*-culture medium (Wang et al., 2019) with modifications. ABC169 is a modified version of Basch medium (Basch, 1981) with the addition of 1% ABAM solution, newborn calf serum (NCS; Sigma-Aldrich, Germany), 200 μM ascorbic acid (Sigma-Aldrich, Germany), and 0.2% V/V human washed red blood cells (10% suspension; Biochrome, Germany). Instead of the original 0.2% V/V porcine cholesterol concentrate used by Wang et al. (Wang et al., 2019), we added low-density lipoprotein (LDL; Trina Bioreactives, Switzerland). As described in the Results section, a concentration of 0.25% V/V LDL turned out to be optimal with respect to worm survival and re-pairing frequency. Worms were kept as couples or separately in six-well plates (Greiner, Germany) at 37°C in 5% CO_2_ in 5 ml medium. Every second day, we changed the medium when worms were monitored over observation periods up to 21 days, depending on the experiments.

Depending on the experimental strategy, we performed re-pairing experiments with pairing-experienced males (bM) and pairing-experienced females (bF) or pairing-unexperienced females (sF), respectively. For some experiments, we separated bM from their female partners at the day of perfusion (= sbM; separated males with previous pairing experience) and kept them in culture for different time-points before re-pairing these males (rbM; repaired bisex males) with either bF or sF. For control purposes, we also used pairing-unexperienced males (single-sex males; sM). For re-pairing, we mixed males and females in ratios of 1.5:1, respectively, which formed stable couples within 72 h under these conditions. These experiments were done in six-well plates (Greiner, Germany) with 5 ml ABC169/LDL (0.25%) medium and 25 worms per plate (15 males, 10 females), and in triplicate (n = 3). When required, we collected worm samples for RNA extraction (five couples each) or we transferred them in fixation buffer (five couples each) for microscopic analysis, as described below.

### 2.3 Confocal laser scanning microscopy

The degree of gonad differentiation in the schistosome female after re-pairing or RNA interference (RNAi) experiments was analyzed by confocal laser scanning microscopy (CLSM). After fixation in AFA (ethanol 66.5%, formaldehyde 3%, and glacial acetic acid 2% in dH_2_O), worms were stained for 30 min with 2.5% carmine red (Certistain H, Merck, Germany), and destained in acidic 70% ethanol. Following dehydration in 70, 90, and 100% ethanol, each for 5 min, worms were mounted in Canada balsam (Merck) (Neves et al., 2005). Images were acquired on a Leica TSC SP5 microscope using a 488-nm He/Ne laser, a 470-nm long-pass filter in reflection mode, and a line average of 4.

### 2.4 Cloning procedures

For cloning of probes for WISH (whole-mount *in situ* hybridization) analysis and target sequences for RNAi (see below), we used the pJC 53.2 plasmid (pJC53.2 was a gift from Jim Collins; Addgene plasmid #26536; http://n2t.net/addgene:26536; RRID : Addgene_26536; Collins et al., 2010), which was cut with *Ahd*I (NEB, UK) for insert integration. As inserts, we amplified DNA/cDNA fragments of Sm*tdc-1* (Smp_135230) and Sm*ddc-1* (Smp_171580), respectively, by gene-specific primer sets (**Supplementary Table 1**) using AccuPrime™ *Taq* DNA Polymerase (high fidelity, Invitrogen/Thermo Fisher Scientific, Germany). For cDNA preparation, we used total RNA of *S. mansoni* as template that was isolated with a commercially available kit following the instructions of the manufacturer (Monarch^®^ Total RNA Miniprep Kit; NEB, UK). We determined total RNA quality and quantity by microfluidic electrophoresis using the Bioanalyzer 2100 system (Agilent Technologies, USA). For reverse transcription (QuantiTect Reverse Transcription Kit; Qiagen, Germany), we used 200 ng of total RNA following the manufacturer’s instruction. PCR products for cloning were separated by agarose gel electrophoresis and then gel-purified using the Monarch^®^ DNA Gel Extraction Kit (NEB, UK). For ligation, we used purified insert DNA/cDNA, 50 ng of *Ahd*I-digested pJC 53.2, and T4 DNA Ligase (NEB, UK). We transformed recombinant plasmids into the DH5a strain of *E. coli* (Thermo Fisher, Germany) and commercially sequenced the generated constructs to verify their integrity (Sanger sequencing; LGC Genomics, Germany).

### 2.5 Quantitative RT-PCR

To assess transcript levels of Sm*tdc-1* and Sm*ddc-1*, we diluted cDNAs 1:7 in RNase-free water before analysis. We performed qRT-PCR experiments with a Rotor-Gene Q cycler (Qiagen, Germany) using the 2x PerfeCTa SYBR Green SuperMix (Quantabio, USA), as described before (Haeberlein et al., 2019). We designed each primer pair (Biolegio; Nijmegen, The Netherlands) for a melting temperature of 60°C and amplicon sizes of 140–200 bp (Primer3Plus; Untergasser et al., 2012; **Supplementary Table 1**) and determined primer efficiencies as described before (Haeberlein et al., 2019). Cycling was done as follows: after an initial denaturation step at 95°C for 3 min, amplification comprised 45 cycles at 95°C for 10 s, 60°C for 15 s, and 72°C, 20 s each. The total reaction volume was 20 μl, containing 400 nM as final primer concentration. We performed all qRT-PCRs in triplicates and determined the expression values with the 2^-ΔΔCt^ method (Livak and Schmittgen, 2001), using Sm*letm1* (Smp_065110) for normalization and the expression in sM as reference point. Sm*letm1* is a proven reference gene for gene expression studies with adult *S. mansoni in vitro* (Haeberlein et al., 2019).

### 2.6 Whole-mount *in situ* hybridization

To localize transcripts of genes, we performed whole-mount *in situ* hybridization (WISH) with adult *S. mansoni* following a published protocol (Collins and Collins, 2017) with minor modifications. For generating hybridization probes, we amplified cDNA inserts cloned into pJC 53.2 plasmids with Q5^®^ High-Fidelity DNA Polymerase (40 U/μl, NEB, UK) using specific primers (**Supplementary Table 1**). Sense transcripts served as control. Digoxigenin (DIG)-labeled probes were generated by *in vitro* transcription with SP6 or T3 RNA polymerases as follows: we mixed 100–500 ng of PCR product, 2 μl of 10× transcription buffer (Roche, Germany), 1 μl T3 or SP6 RNA polymerase (Roche), 2 μl of DIG-NTP mix (10 mM ATP, CTP, and GTP; 7 mM UTP; 3.5 mM DIG-11-UTP (Jena Bioscience, Germany)), 0.6 μl murine RNase inhibitor (40 U/μl, NEB, UK), and RNase-free water to a final volume of 20 μl. The reaction mix was incubated for 16 h at 27°C, followed by the addition of 1 μl of RNase-free DNase I (2 U/μl, NEB, UK) and an incubation for 20 min at 37°C. Probes were stored at -20°C until use.

As worm samples, we used separated *S. mansoni* females, males, and/or couples, which were immobilized by the addition of 0.6 M MgCl_2_ for 1 min while shaking. Next, we fixed the worms for 4 h in 4% formaldehyde dissolved in PBSTX (1× PBS, 0.3% Triton X-100) at room temperature (RT). Fixed worms were dehydrated in 100% methanol and stored at -20°C. Rehydration occurred by incubating the worm samples in 50% methanol dissolved in PBSTX. Samples were bleached for 1 h in bleaching solution (9 ml DEPC H_2_O, 500 μl formamide, 250 μl SSC (20×; pH 7), and 400 μl 30% H_2_O_2_) under direct light. After bleaching, the samples were rinsed with PBSTX. Next, we treated the samples with proteinase K (20 mg/ml, Ambion, AM2546) solution (45 μg/ml PBSTX for males or 20 μg/ml proteinase K for females and couples, respectively) and fixed them in 4% formaldehyde in PBSTX for 15 min. Hybridization was done at 55°C overnight. Subsequent washing and blocking were done as described earlier (Collins et al., 2010). For detection, an anti-DIG-AP (1:2,000, Merck, Germany, 11093274910) antibody was incubated in colorimetric blocking solution (7.5% heat-inactivated horse serum (Merck, Germany, #H1138) in Tris NaCl Tween 20 buffer solution, pH 8.0) overnight at 4°C and developed with nitro blue tetrazolium (Roche, 14799526) and 5-bromo-4-chloro-3′-indolyphosphate (Roche, 13513022). Finally, samples were mounted in 80% glycerol.

### 2.7 Functional analyses by RNAi

We synthesized double-stranded RNA (dsRNA) as previously described with minor modifications (Collins et al., 2010). cDNA templates were amplified by PCR from recombinant pJC 53.2 plasmids using Q5^®^ High-Fidelity DNA Polymerase (40 U/μl, NEB, UK) and gene-specific primers with T7-specific sequence extensions (**Supplementary Table 1**). Based on the amplified templates, dsRNA was generated by *in vitro* transcription with T7 RNA polymerase and the following reaction conditions: 5 μl PCR product (5 μg), 10 μl 10× reaction buffer, 20 μl 25 mM rNTP (NEB, UK), 5 μl T7 RNA polymerase (NEB, UK), 1 μl pyrophosphatase (NEB, UK), inorganic (*E. coli*) (IPP) (NEB, UK), and DEPC water to a final volume of 100 μl. We incubated the reaction mix for 4 h or overnight at 37°C, followed by the addition of 5 μl of RNase-free DNase I (2 U/μl; NEB, UK) and an incubation for 30 min at 37°C; dsRNA was stored at -20°C until needed.

For RNAi, we cultured groups of 15 separated males together with 10 females (either single-sex females or females that had been separated from males for 14 d) in six-well cell-culture plates with 3 ml ABC169/LDL (0.25%) medium at 37°C and 5% CO_2_ to allow pair formation as described above. Depending on the experimental group, we added either Sm*tdc-1* dsRNA (24 μg), or Sm*ddc-1* dsRNA (24 μg), or a combination of both dsRNAs (total: 48 μg), respectively. As negative control, we used untreated worms. All parasites were cultured up to 21 d while we replaced the culture medium and dsRNA every second day. At the same time, we monitored pairing stability, motility, and egg production by bright-field microscopy (Leica, Germany). Egg production was measured by calculating total numbers of normal eggs (per 10 couples) produced over the 21-d period (paired females grown in ABC169/LDL (0.25%) started egg production between days 8 and 10 after re-pairing in culture). After the observation period of 21 d, we collected 15 couples of each experimental group for RNA isolation and qRT-PCR analyses, while we fixed five couples each for CLSM analyses.

### 2.8 *In silico* analyses

We used WormBase Parasite for general sequence information of *S. mansoni* (Howe et al., 2017; https://parasite.wormbase.org; version 7), InterPro/PRINTS to identify protein family fingerprints (Attwood et al., 2003; http://www.ebi.ac.uk/interpro/search/text/PR00800/?page=1#table, accession no. PR00800, YHDCRBOXLASE), and SMART (update 2020) for the identification of sequence motifs (Letunic et al., 2021; http://smart.embl-heidelberg.de/help/smart_about.shtml). Multiple alignments of amino acid sequences were generated with Clustal Omega (Sievers et al., 2011) or, for subsequent phylogenetic analysis, by MEGA11 (Kumar et al., 2021) using MUSCLE (Dress et al., 2008). Maximum-likelihood phylogenies were generated with MEGA11 using the LG substitution model (Le and Gascuel, 2008), and 1,000 replicates during bootstrapping.

### 2.9 Statistical analysis

Data on the transcript levels of Sm*tdc-1* and Sm*ddc-1* are given as mean ± SEM. Significant differences as determined by one-way ANOVA (GraphPad Prism 7) and Tukey’s test for multiple comparisons (Midway et al., 2020) are indicated by *p < 0.05, **p < 0.01, and ***p < 0.001. We considered all p-values < 0.05 as significant.

## 3 Results

### 3.1 Improved culture conditions assured optimal re-pairing rates of *S. mansoni in vitro*

One of the challenges of the *in vitro* maintenance of *S. mansoni* for pairing experiments is to achieve high efficiency re-pairing and to ensure pairing stability and egg production over time. Progress in this field has been made by developing the *in vitro*-culture medium ABC169 that supports female development following pairing and finally leads to enhanced egg production over time (Wang et al., 2019). This is an improvement compared with the formerly used Basch medium BM169 (Basch, 1981) and commercial M199 medium. Due to restricted availability, however, we replaced the porcine cholesterol originally used as additive for ABC169 (Wang et al., 2019). Instead, we used human LDL, which we also considered to better mimic the supply by the human host environment. Starting with equal numbers of males and females (five per sex) and different concentrations of LDL (0%–2%), we found 0.125%–0.5% LDL (V/V) as most suitable with respect to re-pairing efficiency and worm vitality. A concentration of 2% LDL caused the death of worms within 3 days of culture. Furthermore, compared with the standard M199 medium for schistosome culture, ABC169/LDL (0.25%) medium generally ensured higher re-pairing efficiencies, which were best with male/female ratios of 6:2 or 15:10 in 5 ml medium leading to 100% re-pairing within 48–72 h (**Supplementary Figure S1**).

### 3.2 Pairing induced oocyte differentiation in single-sex females within 11 days *in vitro*

Using the improved culture conditions, we investigated the time period needed for single-sex females to start oocyte differentiation upon paring *in vitro*. For pairing experiments, we collected pairing-experienced males from bisex infections by manually separating these males from their female partners right after perfusion. We kept separated males for 1 day as male-only populations *in vitro* before single-sex females were added for re-pairing in a male/female ratio of 15:10. Re-pairing occurred within 2–3 d. To investigate oocyte differentiation as a biological indicator for female sexual maturation, we monitored reformed couples after at days 3, 6, 11, and 17 of the experiment before we stopped it by fixing couples of each time point for subsequent morphological analyses. By CLSM, we observed differentiated oocytes in females at day 11 of the experiment (**Supplementary Figure S2**). This indicated that oocyte differentiation started between days 6 and 11 under these *in vitro* culture conditions. Therefore, we used these time-points in subsequent *in vitro* culture experiments addressing the role of decarboxylase genes.

### 3.3 Smp_135230 and Smp_171580 are pairing-dependently transcribed genes in male *S. mansoni*

**Figure 1:**
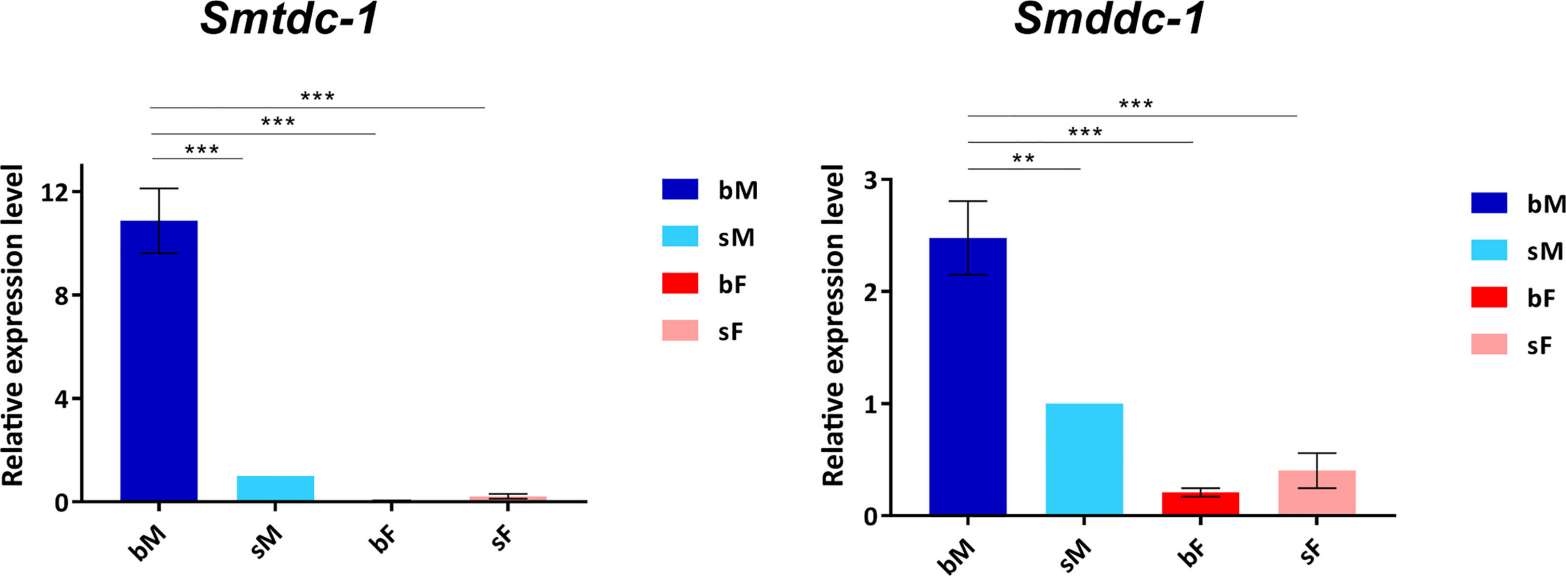
qRT-PCR analysis comparing transcript levels of Sm*tdc*-1 and Sm*ddc*-1 in adults. We analyzed relative transcript levels of Sm*tdc*-1 and Sm*ddc*-1 using the 2^-ΔΔCt^ method. Data are representatives of the mean ± SEM of three separate experiments. For normalization, we used Sm*letm1* (Smp_065110), which was shown before to be a reliable reference for gene expression analysis of *S. mansoni in vitro* (Haeberlein et al., 2019), and the sM data as reference point. Statistical analysis was done by one-way analysis of variance (ANOVA) and Tukey’s test for multiple comparisons, as indicated: **p < 0.01, and ***p < 0.001.

**Figure 2:**
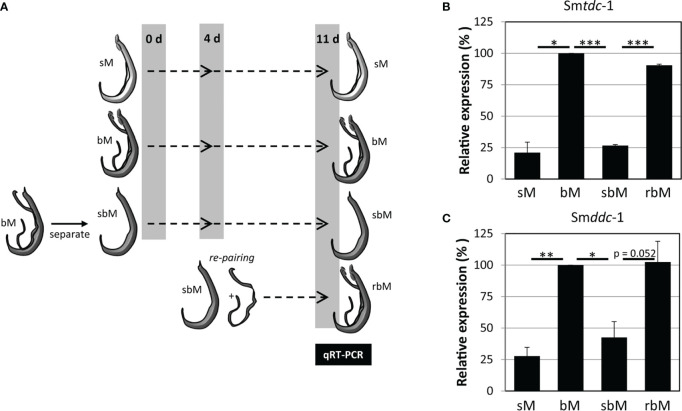
Pairing controls on–off switching of Sm*tdc-1* and Sm*ddc-1* expression in *S. mansoni* males. **(A)** Ahead of molecular analysis, *S. mansoni* males with varying pairing states were cultured for 11 days: unpaired, single-sex males (sM), paired bisex males that were separated from their female partners at day 11 (bM), bM that were temporarily separated from females and kept isolated from females for 11 days (sbM) or re-paired with females at day 4 (rbM). **(B)** The relative expression levels of Sm*tdc-1* (Smp_135230) and **(C)** Sm*ddc-1* (Smp_171580) in males at day 11 of the experiment (for this, males of the bM and rbM groups were separated from females) were quantified by qRT-PCR and normalized to Sm*letm1*, a validated reference gene for expression analyses of *S. mansoni* genes under *in vitro*-culture conditions (Haeberlein et al., 2019). Data are expressed relative to bM (set to 100%). A summary of three independent *in vitro* culture experiments is shown with standard errors of the mean. Statistical analysis was done by one-way analysis of variance (ANOVA) and Tukey’s test for multiple comparisons. Significant differences are indicated with *p < 0.05, **p < 0.01, and ***p < 0.001.

Previous transcriptomics studies using SuperSAGE and microarray analyses with male *S. mansoni* identified Smp_135230 and Smp_171580 as sex-specifically or sex-preferentially as well as pairing-dependently transcribed genes (Leutner et al., 2013). RNA-seq analyses confirmed these findings and exhibited a strong expression bias of bM > sM for both genes, whereas nearly no expression occurred in females except for Smp_171580, which showed a low transcript level in sF but nearly no transcripts in bF (Lu et al., 2016; Lu et al., 2017) (**Supplementary Figure S3**). To substantiate these preliminary transcript profiles, we performed qRT-PCR analysis comparing the transcript levels of both genes in males versus females, and depending on the pairing status. The result finally confirmed nearly male-specific (Smp_135230) or male-preferential (Smp_171580) expression profiles with higher transcript abundance of both genes in bM than in sM (**Figure 1**). Next, we quantified the influence of pairing on the on/off status of both genes following a previously published approach (Haeberlein et al., 2019). To this end, sM and bM groups were cultured for 11 d *in vitro*. A further bM group consisted of pairing-experienced males that were separated from their female partners immediately after perfusion. We continued to culture these separated males as single males (sbM group) in parallel to the sM and bM groups. After 4 days, we took half of the sbM group for coculture with females. Re-pairing occurred within 48 h, and as couples we cultured these worms for another 7 days. At day 11, we stopped the experiment, separated the couples, and extracted RNA from the males of all groups for qRT-PCR analyses. For both genes, we found a strong pairing influence on their transcript levels (**Figure 2**). Over a culture period of 11 days, the transcript levels of both genes were high in bM and low in sM. Upon separation, however, the transcript levels of both genes decreased in sbM within 4 days. Following re-pairing of sbM with females, within 5–7 days the transcript levels of both genes increased again to the level of the bM group.

### 3.4 Structural and phylogenetic analyses indicated roles for Smp_135230 and Smp_171580 as L-tyrosine and DOPA decarboxylases, respectively

**Figure 3:**
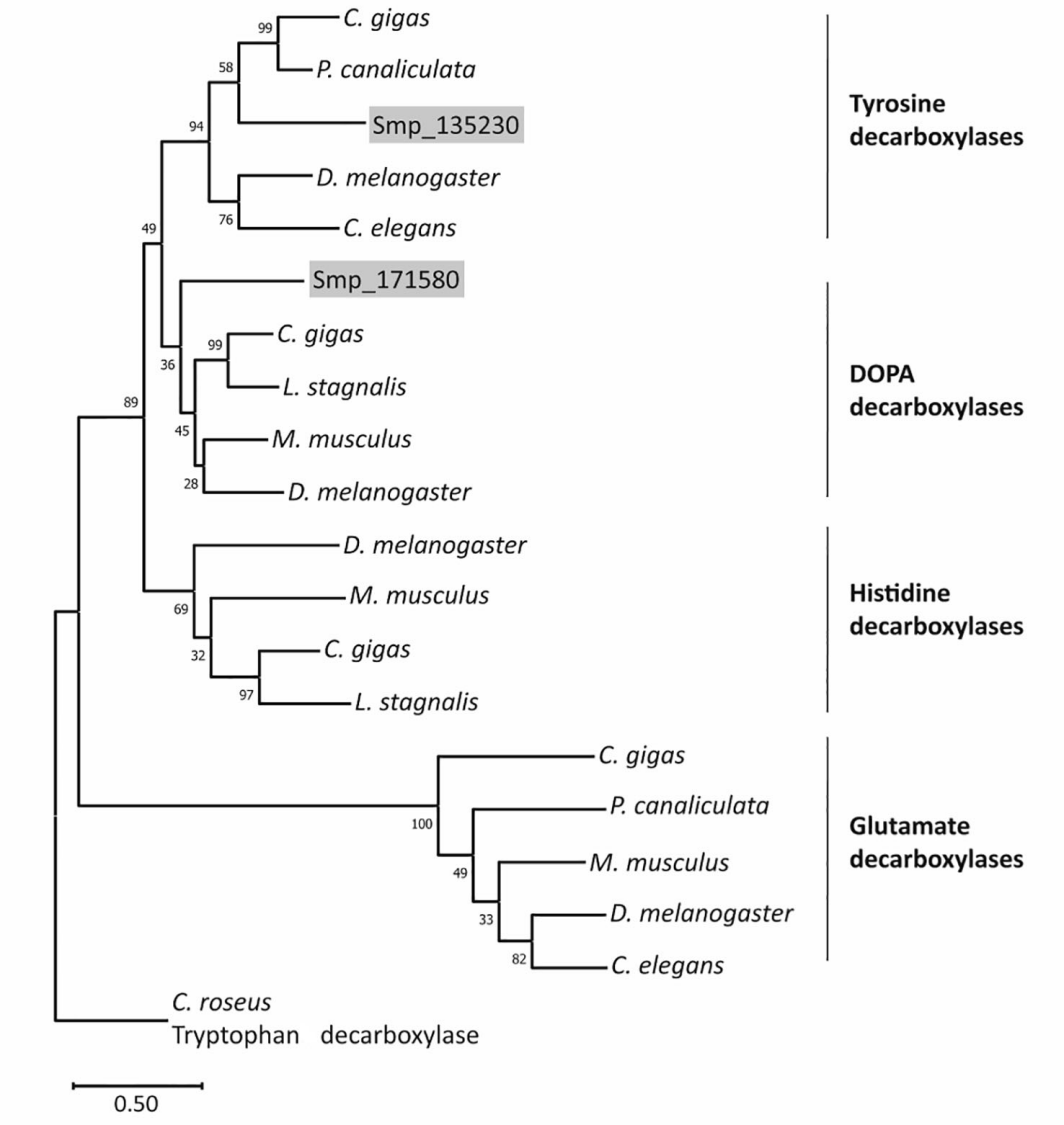
Phylogenetic analysis arranges SmTDC-1 and SmDDC-1 of *S. mansoni* in different decarboxylase clades. The maximum-likelihood phylogram included aa sequences of decarboxylases of selected animals in relation to SmTDC-1 (Smp_135230) and SmDDC-1 (Smp_171580). The resolved families of DOPA, histidine, tyrosine, glutamate decarboxylases are marked. Bootstrap values are shown at each node; scale bar = the average number of substitutions per site along each branch. Species included *Mus musculus* (mouse), *Drosophila melanogaster* (fly), *Crassostrea gigas* (oyster), *Pomacea canaliculata* (snail), *Lymnaea stagnalis* (snail), and *Caenorhabditis elegans* (nematode). *Catharanthus roseus* (periwinkle plant) was used as outgroup. For sequences and accession numbers (see also **Supplementary Figure S3B**).

In WormBase ParaSite (Howe et al., 2017), both genes are annotated as DOPA decarboxylase orthologs. Sequence analyses revealed that each gene contains a characteristic pyridoxal domain (Ishii et al., 1996). According to SPRNIT database information, pyridoxal-dependent group II decarboxylases share regions of similarity that are represented by nine typical but variable sequence motifs (accession PR00800; Jackson, 1990; Sandmeier et al., 1994; Attwood et al., 2003), all of which occur in both protein sequences (**Supplementary Figure S4A**). At a specific and conserved position within motif 8, one aa differentiates between tyrosine decarboxylases (S, in Smp_135230) and DOPA decarboxylases (G, in Smp_171580) (Torrens-Spence et al., 2014). To provide further evidence for the assumption that Smp_135230 represents a L**-**tyrosine decarboxylases (TDC) and Smp_171580 a DOPA decarboxylase (DDC; also known as aromatic L-amino acid decarboxylase AADC), we performed phylogenetic analyses (**Figure 3**; **Supplementary Figure S4B**), which grouped Smp_135230 to the tyrosine decarboxylase clade (Sm*tdc-1*) and Smp_171580 to the DOPA decarboxylase clade (Sm*ddc-1*), respectively. Sequence alignments of the selected orthologs additionally exhibited high homology among these sequences including the presence of highly conserved and characteristic aa residues (**Supplementary Figures 4C, D**). These results prompted us to rename both genes, Sm*tdc-1* for Smp_135230 and Sm*ddc-1* for Smp_171580.

### 3.5 Sm*tdc-1* and Sm*ddc-1* transcripts exhibited distinct localization patterns probably including neurons

**Figure 4:**
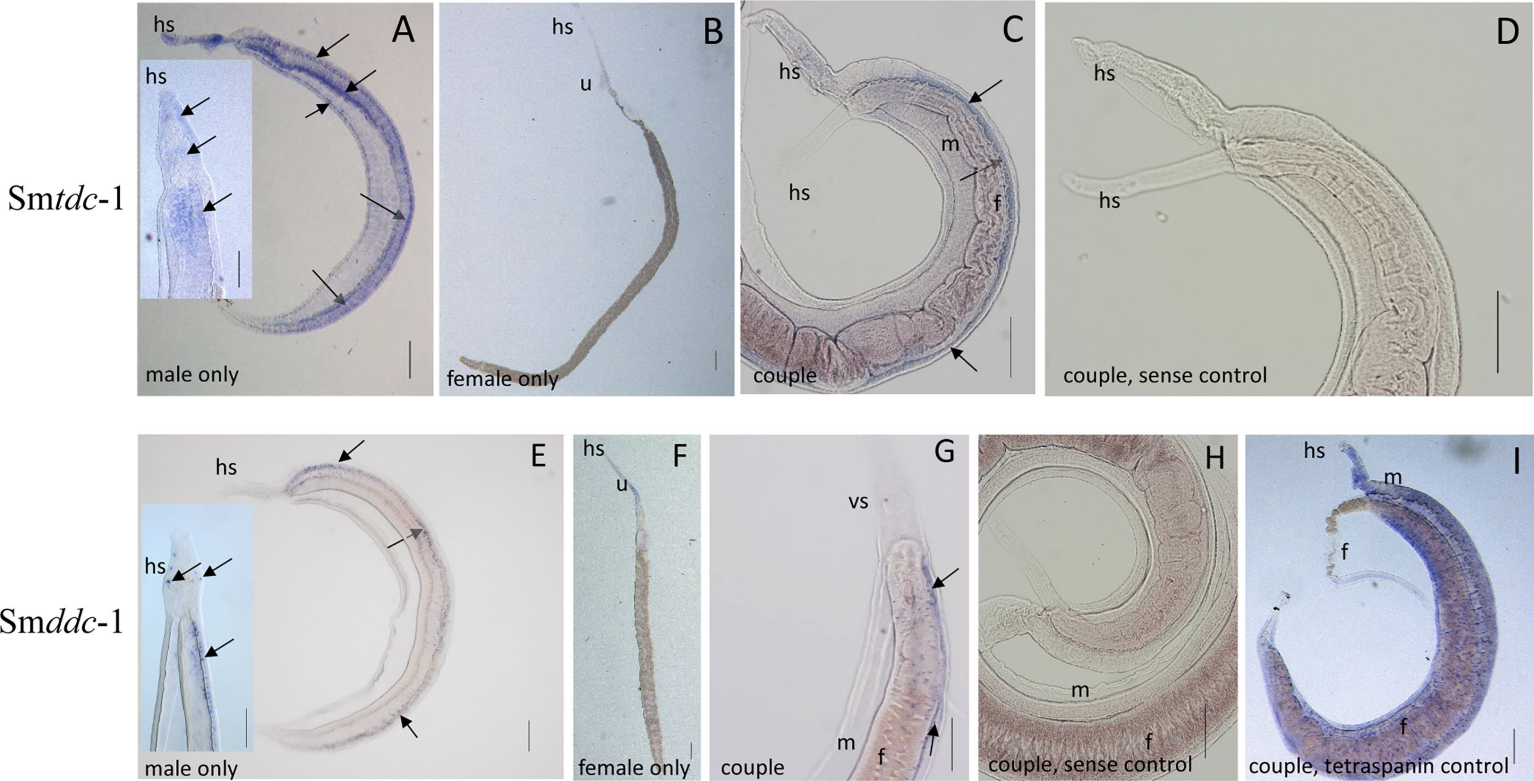
WISH analyses showing neuronal patterns of Sm*tdc-1* and Sm*ddc-1* transcripts. For localization of Sm*tdc-1* and Sm*ddc-1*, we designed antisense and sense (control) probes for each gene from regions that showed no sequence homology to the other gene (**Supplementary Table 1**). WISH was performed as previously described (Collins and Collins, 2017). Hybridization was performed with couples, and for documentation, some couples were separated before microscopic analysis **(A, B, E, F)**, whereas other couples remained intact for analysis **(C, D, G, H)**. With the antisense probes for Sm*tdc-1* and Sm*ddc-1*, dominant signals occurred in males (**A, C, E, G**; blue arrows) but only weak signals in females **(B, F)**, as expected (Lu et al., 2016). Sm*tdc-1* transcripts occurred in a regular pattern along the male body, which points to neuronal cells, but weakly also in the parenchyma and the tegumental area (**A**, including magnified inset). Sm*ddc-1* transcripts occurred in a more specific, dotted pattern along the male body, which might also point to neuronal cells (**E**, including magnified inset). Many of the cells seemed to occur at the ventral surface of the male (**A, C, E,** dashed blue arrows). As negative controls, we used sense probes of both genes that showed no signals upon hybridization **(D, H)**. As positive control **(I)**, we used a probe detecting Sm*tsp-2* of *S. mansoni*, a tetraspanin gene with known expression pattern along the tegumental surface area of *S. mansoni* (Tran et al., 2010). f, female; m, male; hs, head sucker; u, uterus. Scale bars, 200 µm.

Next, we performed WISH to localize Sm*tdc-1* and Sm*ddc-1* transcripts in adult *S. mansoni* with gene-specific antisense probes. As expected, strong signals representing transcripts of both genes occurred in males but not in females (**Figure 4**). Within males, we detected transcripts of both genes mainly at the ventral side of the male body, which forms the gynecophoral canal. Although we cannot exclude the participation of other tissues, the occurrence of dotted signals in a string-like order along the middle axis of the male body suggested a neuronal expression pattern. This interpretation is substantiated by data of a recent single-cell analyses that confirmed Sm*tdc-1* and Sm*ddc-1* transcripts in different cell populations with a dominance for neuronal cells, especially the neuron-2 cluster (Wendt et al., 2020; **Supplementary Figure S5**). In females, we observed only weak signals for Sm*tdc-1* and Sm*ddc-1* along the uterus. As controls, we used sense probes of both genes that showed no signals. Moreover, as positive control, we used a probe detecting transcripts of the tetraspanin gene Sm*tsp-2* of *S. mansoni*. Its transcript occurrence along the tegumental area was shown previously (Tran et al., 2010), which was confirmed here showing a ubiquitous surface expression pattern.

### 3.6 Knocking down Sm*tdc-1* and Sm*ddc-1* transcripts caused regression of reproduction-associated processes in females

**Figure 5:**
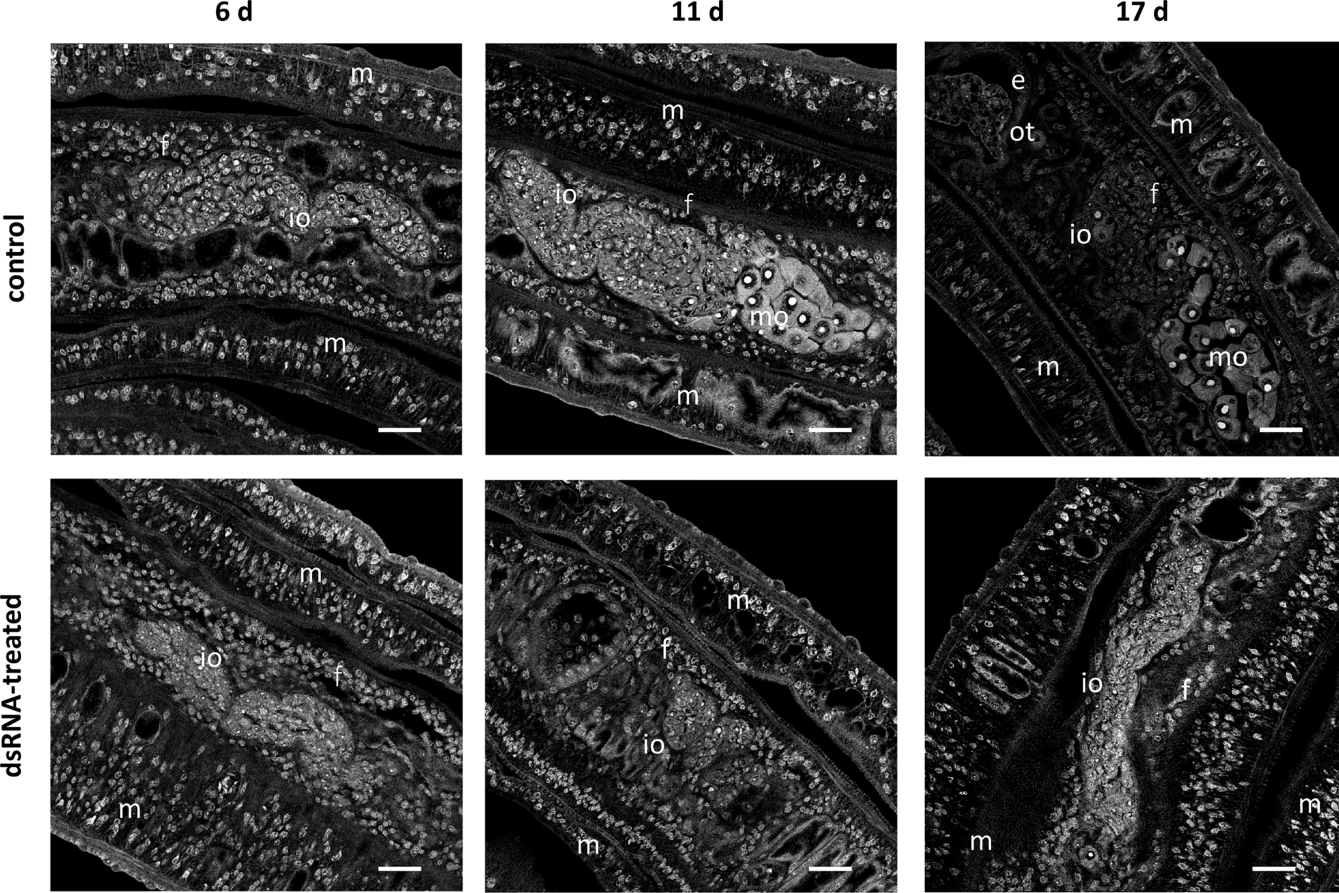
RNAi against Sm*tdc-1* and Sm*ddc-1* impedes oocyte maturation upon re-pairing of previously separated females. RNAi was performed against Sm*tdc-1* and Sm*ddc-1* in group 1 dedifferentiated females, which were obtained by separating *S. mansoni* couples immediately after perfusion. As single females, they were left for 7 d in culture before they were re-paired with males again. DsRNA against Sm*tdc-1* and Sm*ddc-1* was added 4 days before re-pairing, and the couples were incubated over a 21-d period. At days 6, 11, and 17, couples were removed from the culture dishes, fixed, stained with carmine red, and used for CLSM. After 6 d, only immature oocytes (io) occurred in paired females (f) in both the control and treatment groups. This changed after 11 and 17 d. At both time points, we found mature oocytes (mo) only in the control but not in the treatment group. m, male; e, egg (inside the ootype); scale bars, 25 µm.

To unravel the biological functions of Sm*tdc-1* and Sm*ddc-1*, we performed different series of RNAi experiments against both genes, individually and in combination. Furthermore, we studied the potential pairing-associated role of these genes by pairing experiments using female *S. mansoni* with varying pairing and differentiation history. In a first experimental approach, we used dedifferentiated females (group 1). To the end, sexually mature females, bF (obtained from bs infections), were separated from males immediately after perfusion. We left these separated bF as female-only populations in culture for 7 d to induce a sexual dedifferentiation status of these females. CLSM analysis showed the loss of mature oocytes already 6 d of separation of females from males, which indicates that around 1 week is sufficient to interrupt oocyte maturation in females with previous pairing experience *in vitro*. (**Figure 5**, day 6). Furthermore, no more eggs were produced at that time point (data not shown). Next, we re-paired these females with males. To control for the reversibility of the de-differentiation process, we checked for oocyte maturation and egg production after re-pairing. Both resumed within 11 days as demonstrated by the occurrence of mature oocytes as well as eggs within the ootype (**Figure 5**, control, days 11 and 17), and eggs released in the culture medium (data not shown).

In a separate pairing approach, we used females in a naturally undifferentiated state, i.e., without previous pairing experience (obtained by ss infection) (group 2). These single-sex females (sF) have a sexually immature character, and as such, no vitellarium and an incompletely developed ovary that is exclusively equipped with stem-cell like oogonia (Popiel and Basch, 1984a; Beckmann et al., 2010). With both pairing groups, we subsequently performed physiological (motility, vitality, pairing stability, egg production) and morphological (bright-field microscopy and CLSM) assays *in vitro* to monitor female reproductive activity and development upon RNAi.

For studying RNAi effects on pairing stability, males and group 1 females were treated with dsRNA starting 4 days prior to re-pairing. Over the next 21 days, dsRNA treatment was maintained, and re-pairing frequency, egg production, motility, and vitality were monitored on a daily basis (n = 6). After this period, the experiment was finished by separating reformed couples to extract RNA from males. By qRT-PCR, we determined knockdown efficiencies, which varied between 50% and 90% depending on the gene (**Supplementary Figure S6A**). RNAi against either Sm*tdc-1* or Sm*ddc-1* had no influence on motility and vitality (not shown). However, combining dsRNAs to target both genes in parallel showed effects. First, we observed a weak trend of reduced re-pairing frequency during the first days (d1–d7) of re-pairing compared with single-gene RNAi or the (untreated) control group (**Supplementary Figure S6B**). Second, analyzing egg production over the complete observation period of 21 d, we found significantly lower numbers of eggs in the treatment groups compared with untreated control couples (**Supplementary Figure S6C**).

In addition to these physiological effects, we investigated potential morphological effects by CLSM using worms of both re-pairing approaches. After re-pairing group 1 females with males, we observed a remarkable RNAi effect. In contrast to controls (untreated), dsRNA-treated, re-paired females failed to reach the complete differentiation status of the ovary. Treatment with either Sm*tdc-1* or Sm*ddc-1* led to a decrease in the number of mature oocytes in both cases (**Supplementary Figure S6D**) over the observation period of 21 d. This phenotype enhanced upon combined treatment with dsRNA against Sm*tdc-1* and Sm*ddc-1* (**Figure 5**). After 6 d, we found no difference between dsRNA-treated and non-treated control females of the re-pairing group 1. However, after d11 and d17, clear differences were observed. In the control group, we detected mature oocytes in re-paired females at both time points, d11 and d17. However, no mature oocyte occurred in the dsRNA treatment group at these time points.

**Figure 6:**
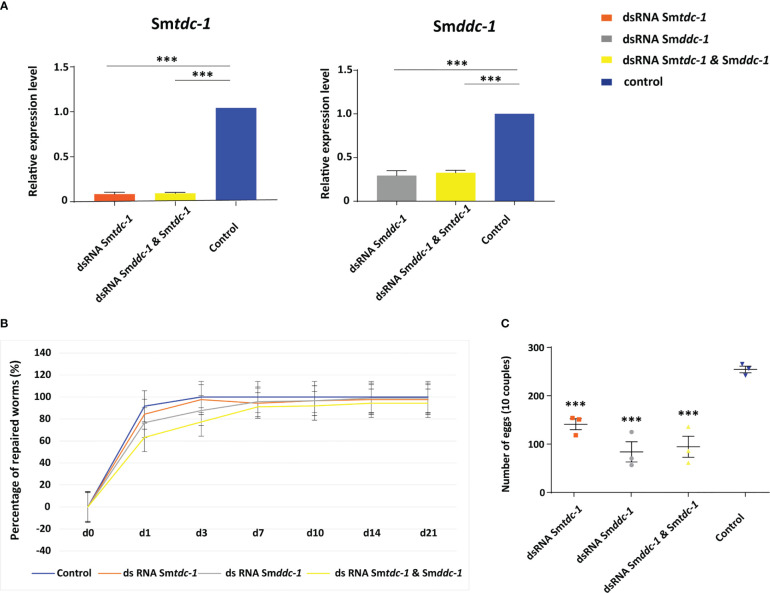
RNAi against Sm*tdc-1* and Sm*ddc-1* in first-time paired females reduces egg production. RNAi was performed against Sm*tdc-1* and Sm*ddc-1* in group 2 virgin females (without previous pairing experience). **(A)**, following RNAi, transcript levels in males were reduced down to 40%–10% as determined by qRT-PCR using Sm*letm1* (Smp_065110). **(B)**, the diagram shows the summary of re-pairing frequencies of *S. mansoni* males with group 2 females *in vitro* following RNAi with dsRNA targeting Sm*tdc-1* (Smp_135230) and Sm*ddc-1* (Smp_171580), either individually (gray and orange lines) or in combination (yellow line; n = 3; each n with 10 worms of both genders). Control, untreated worms maintained *in vitro* for re-pairing under the same conditions (blue line; n = 3; each n with 10 worms of both genders). No statistically significant differences were observed. **(C)**, summary of egg counts over the observation period of 21 d. Significantly lower numbers of eggs occurred in the treatment groups compared with the control (n = 3). **(A, C)**, statistics were done by one-way ANOVA and Tukey’s test for multiple comparisons. Data show the mean ± SEM of three separate experiments, and statistically significant differences are shown as ***p < 0.001.

**Figure 7:**
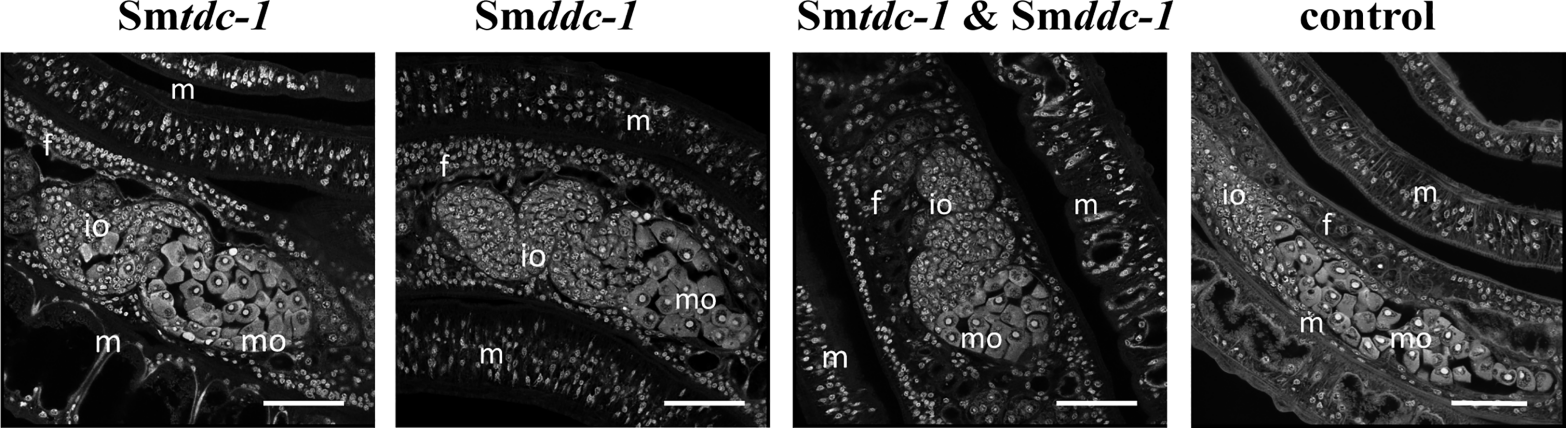
RNAi against Sm*tdc-1* and Sm*ddc-1* decreases the number of mature oocytes of first-time paired females. CLSM analyses of group 2 females showed lower numbers of differentiated oocytes in the treatment groups (RNAi against Sm*tdc-1 and* Sm*ddc-1*, and both in the combination) compared with the respective controls at day 21 after re-pairing. f, female; m, male; mo, mature oocytes;, io, immature oocytes; scale bars 50 µm.

We performed an analogous approach with group 2 females (sF), which we paired with males *in vitro* directly upon perfusion, and which we treated with dsRNA for 21 days. By qRT-PCR, we determined knockdown efficiencies that were in a similar range of 60%–90% (**Figure 6A**), as reported for group 1 females. Pairing occurred within 2 days. Also for paired females, RNAi against the target genes showed no obvious effects on motility and vitality. Similar to group 1 females, combining dsRNAs for treatment resulted in a trend of reduced re-pairing frequency during the first 7–10 days of the observation period (**Figure 6B**). Determining egg production over the complete observation period of 21 days showed significantly lower numbers of eggs in the treatment groups compared with untreated control (**Figure 6C**). Correspondingly, compared with the control, also in group 2 female treatment with either Sm*tdc-1* or Sm*ddc-1* or a combination of both led to a decrease in the number of mature oocytes in each case as shown by CLSM (**Figure 7**). All obtained data for group 2 females were similar to the results obtained with group 1 females.

Finally, we investigated the morphology of the vitellarium of group 1 and 2 females following pairing with males and treatment with dsRNA against Sm*tdc-1* or Sm*ddc-1*, or the combination of both. We detected no difference between the dsRNA-treatment groups or untreated controls by CLSM (not shown). In the same experiment, we also investigated RNAi effects on the male gonad and found no effects on testes size, occurrence of spermatogonia, or differentiated sperm (**Supplementary Figure S7**).

## 4 Discussion

Research activities of the last decade have impressively demonstrated the enormous diversity of communication principles among biological communities and between individual organisms. This includes parasites and their fascinating wealth of strategies to communicate with their environment (Roditi, 2016). Among these are schistosomes, the only mammalian flatworms that have evolved separate sexes. Trancriptomics data obtained from various studies have provided convincing support for a tremendous impact of pairing on the expressed gene repertoire of schistosome females and their reproductive biology (Fitzpatrick and Hoffmann, 2006; Cai et al., 2016; Lu et al., 2016; Wang et al., 2017). However, our knowledge about the bidirectional molecular communication of the genders is limited (Lu et al., 2019). The contribution of the male seems not restricted to nutritional support and muscular strength to ship the paired female, which resides inside the male’s gynecophoral canal, to the final host destination. Male–female interaction appears more complex and involves a flow of information between the partners to direct transcriptional processes in females but also in males, as shown for *S. mansoni* (Fitzpatrick and Hoffmann, 2006; Lu et al., 2016) and *S. japonicum* (Cai et al., 2016). Results of former studies indicated that pairing-experienced *S. mansoni* males induce mitogenic activity in paired females faster than pairing-unexperienced males do (Den Hollander and Erasmus, 1985). This supports the idea of a kind of molecular competence a male has to achieve upon a first, initial pairing contact before it is able to induce female sexual maturation (Lu et al., 2019). Against this background, it is of interest to investigate genes that show a bM > sM expression bias as they may belong to the repertoire of competence factors needed by the male to fulfill its partner role. Among these genes of interest were Sm*tdc-1* or Sm*ddc-1*, whose elevated transcript levels in bM versus sM were correspondingly found in SuperSAGE and microarray experiments (Leutner et al., 2013), RNA-seq (Lu et al., 2016), and qRT-PCR analyses of a former (Haeberlein et al., 2019) and this study.

Sequence analyses indicated that Smp_135230 is a L**-**tyrosine decarboxylase (Sm*tdc-1*) and Smp_171580 a DOPA decarboxylase (Sm*ddc-1*), which was supported by phylogenetic analysis. To a large extent, the WISH results of Sm*tdc-1* and Sm*ddc-1* corresponded to the qRT-PCR data with respect to male-specific/preferential transcript occurrence. The signal pattern upon WISH suggests that both genes are transcribed at the ventral surface area of the male, the gynecophoral canal. This interpretation agrees with *in situ* hybridization results of a previous study of a Smp_171580 ortholog of *S. japonicum*, SjAADC (CCD58963.1; EWB00_004068.2) (Wang et al., 2017), transcripts of which have been localized at the male gynecophoral canal. The stripe-like, spotty signal pattern observed in our WISH analysis indicated the occurrence of Sm*tdc-1* and Sm*ddc-1* transcripts in neuronal cells. Indeed, in the first single-cell atlas for *S. mansoni*, highest transcript levels of both genes were found in the neuron-2 cluster (Wendt et al., 2020; **Supplementary Figure S5**), which according to our analysis may be represented by neuronal cells at the ventral side of the male along the gynecophoral canal. Interestingly, a similar pattern of ventral cells was recently observed in WISH analyses of Sm*nrps* ^+^ cells (nrps, non-ribosomal peptides), which were also identified as part of the neuron-2 cluster. Being ciliated, these cells may fulfil sensory functions, which favors the idea that they are directly involved in male–female communication (Chen et al., 2022). Transcript profiling of the Sj*aadc* gene showed an increase of expression in males between days 14 and 18 of pairing, whereas the transcript level remained low and pairing-independent in females. This was interpreted as potential function of this gene for motivation, movement, and motility to maintain copulatory activity of male *S. japonicum* (Wang et al., 2017). According to a meta-analysis of gene expression profiles during development in *S. mansoni* (Lu et al., 2018), the transcript level of Sm*tdc-1* showed a similar pattern being upregulated in the juvenile stage between days 21 and 28, whereas the transcript level of Sm*ddc-1* increased already in 24 h schistosomula. In both cases, the transcript levels of females decreased after pairing in females but remained high in their male partners. Our functional analyses of Sm*tdc-1* and Sm*ddc-1* by RNAi exhibited complementing, reproduction-associated phenotypes in the female. In dsRNA-treated paired females, egg production was significantly reduced in both groups of females, pairing-experienced females that were separated and later re-paired, and single-sex females paired for the first time. In both groups, no significant effect on pairing stability was observed during the treatment period. Subsequent morphological analyses by CLSM revealed a substantial decrease of mature oocytes, correspondingly in both female groups. This phenotype was observed in single-gene knockdown experiments but enhanced upon double knockdown. The latter finding suggests that both genes fulfill complementary or additive roles in oocyte maturation.

Of note, these data were obtained using a modified media formulation and a male:female ratio of 1.5–3:1 to optimize re-pairing frequency *in vitro*, which appeared to be supported by human LDL. Under these conditions, we obtained up to 100% re-pairing frequency within 2–3 days. Furthermore, separation and re-pairing experiments were accompanied by CLSM analyses and showed that a separation period from males for 6 days can be sufficient for dedifferentiation and the loss of capacity to generate mature oocytes in (previously paired) females. This finding, however, may vary from worm batch to worm batch, from the culture conditions (including serum source and batch), and from the schistosome strain used. Therefore, female gonad dedifferentiation following the separation of couples should be monitored by CLSM.

Our findings correspond to former findings of BrdU (5-bromo-2′-deoxyuridine) incorporation experiments that demonstrated a tremendous decline of mitogenic activity in pairing-experienced females within 2–5 days of separation from their male partners and a resumption of mitogenic activity after 7 days of re-pairing (Knobloch et al., 2006). Since the induction of mitogenic activity in females is the first process induced by pairing and a prerequisite for subsequent differentiation processes in the female gonads (Den Hollander and Erasmus, 1985), our results meet the expectations. Indeed, oocyte maturation and egg production resumed in our hands within 11 days following re-pairing. Beyond that, the outlined *in vitro* pairing approach with (previously) pairing-experienced and (6 d) separated females, which were reset to a virgin-like female status this way, opens an alternative strategy for experiments with a focus on female–gonad differentiation in case of limited access to virgin females from single-sex infections.

The results of the RNAi experiments suggest essential roles of Sm*tdc-1* and Sm*ddc-1* for male–female interaction and the pairing-dependent gonad development and differentiation of mature oocytes. As male-specifically (Sm*tdc-1*) or male-preferentially (Sm*ddc-1*) and strict pairing-dependently expressed genes, they may represent competence factors of males, whose existence has previously been proposed (Lu et al., 2019). L-Tyrosine decarboxylases and the aromatic-L-amino-acid decarboxylase are potentially involved in the synthesis of biogenic amines (BAs) like dopamine, serotonin, histamine, and tryptamine, which can act as neurotransmitters. Former studies in schistosomes indicated the presence of biosynthetic pathways for the major BA classes, the presence of BA receptors, and the role of BAs as major modulators of neuromuscular function controlling movement, attachment, and behavior (Pax et al., 1984; Ribeiro et al., 2012). Already in the 1980s, the presence of serotonin was shown in sensory nerve endings located near the surface of the parasite (Gustafsson, 1987). Finally, a bioinformatics study suggested the existence of probably 24 BA receptors in *S. mansoni* (Zamanian et al., 2011). Altogether, these results suggest fundamental roles of BAs for schistosome biology.

From our data, it appears tempting to speculate that BAs may also be involved in the male-dependent control of female sexual maturation. In the absence of a tryptophan decarboxylase-annotated ortholog in *S. mansoni* (according to WormBase ParaSite), one of the putative roles of the aromatic L-amino acid/DOPA decarboxylase SmDDC-1 could be to provide the tryptamine substrate for the non-ribosomal peptide synthetase, an enzyme that is expressed in males upon pairing to generate β-alanyl-tryptamine (BATT). This pheromone-like dipeptide was recently shown to stimulate female sexual maturation including egg production in single-sex females, independent of the male’s presence (Chen et al., 2022). Compared with naturally paired females, however, egg number and quality as well as ovary differentiation were inferior in BATT-treated single-sex females. This indicates the contribution of further factors acting in conjunction with BATT to fully accomplish the sexual maturation of female schistosomes.

## Data availability statement

The datasets presented in this study can be found in online repositories. The names of the repository/repositories and accession number(s) can be found in the article/**Supplementary Material**.

## Ethics statement

The animal study was reviewed and approved by Regional Council (Regierungspraesidium) Giessen (V54-19 c 20/15 h 02 GI 18/10 Nr. A 26/2018).

## Author contributions

Designing research studies: CG. Funding acquisition: CG. Conducting experiments and acquiring the data: XL, OW, SH. Analyzing the data: XL, OW, SH, CG. Writing manuscript: XL, CG. Reviewing the manuscript: XL, OW, SH, CG. All authors contributed to the article and approved the submitted version.
