# Supplementary material for: Molecular characterization of Smtdc-1 and Smddc-1 discloses roles as male-competence factors for the sexual maturation of Schistosoma mansoni females
Source: Front Cell Infect Microbiol. 2023 May 26;13:1173557. doi: 10.3389/fcimb.2023.1173557 (PMC10252128; doi:10.3389/fcimb.2023.1173557)
Supplement: Supplementary file 1 [file DataSheet_1.zip › Supplementary Material .docx]

**Supplementary Material**

**Supplementary Figure S1:**

**Effect of different LDL concentrations and different media on re-pairing and vitality**

**A. Effect of different LDL concentrations**

| **Re-paired worms** | **2% LDL** | **1% LDL** | **0.5% LDL** | **0.25% LDL** | **0.125% LDL** | **0% LDL** |
| --- | --- | --- | --- | --- | --- | --- |
| **Day 2** | **2.7%** | **67.0%** | **88.0%** | **88.0%** | **82.0%** | **69.0%** |
| **Day 4** | **0** | **42.0%** | **85.0%** | **90.0%** | **82.0%** | **76.0%** |
| **Dead worms** | **2% LDL** | **1% LDL** | **0.5% LDL** | **0.25% LDL** | **0.125% LDL** | **0% LDL** |
| **Day 2** | **14.5%** | **0** | **0** | **0** | **0** | **0** |
| **Day 4** | **50.9%** | **5%** | **0** | **0** | **0** | **0** |

**B. Tests with different media and varying combinations of males and females for re-pairing efficiency**

**ABC169/LDL(0,25%) M199**

|  | **4:4** | **3:9** | **6:2** | **15:10** | **4:4** | **3:9** | **6:2** | **15:10** |
| --- | --- | --- | --- | --- | --- | --- | --- | --- |
| **Day 1** | **63.90%** | **81.48%** | **66.67%** | **77.50%** | **25.00%** | **62.96%** | **50.00%** | **50.00%** |
| **Day 2** | **83.33%** | **96.29%** | **100.00%** | **92.50%** | **61.11%** | **81.48%** | **61.11%** | **70.00%** |
| **Day 3** | **94.44%** | **96.29%** | **100.00%** | **100.00%** | **52.78%** | **77.77%** | **50.00%** | **83.33%** |

Suppl. Fig. S1: **A**, groups of each 5 males and 5 females were used to investigate the effects of different LDL concentrations on re-pairing and vitality over a three-day observation period *in* *vitro*. LDL in concentrations of 0.125% - 0.5% turned out to be most suitable for re-pairing. At the same time, this range had no negative effect on worm vitality, which was negatively influenced at a concentration of 2% LDL (mean of n=11). **B**, groups with different numbers of males (blue numbers) and females (red numbers) as starting population were investigated for re-pairing efficiency. A comparison to standard M199 medium, which is one of the frequently used media for the *in vitro* maintenance of *S. mansoni*, revealed a better performance of the ABC169/LDL(0,25%) medium with respect to re-pairing efficiency (mean of n=3). Ratios of 6:2 or 15:10 appeared most suitable to achieve 100% re-pairing within 3 d *in vitro* when ABC169/LDL(0,25%) was used. Blue digits: number of males; red digits: number of females.

**Supplementary Figure S2:**

**Single-sex females showed oocyte differentiation within 11 days after pairing *in vitro***


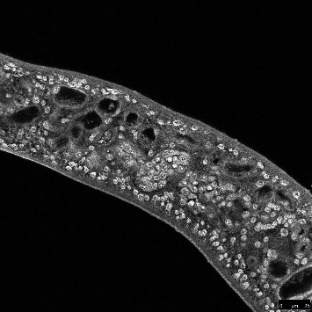


3 d

11 d


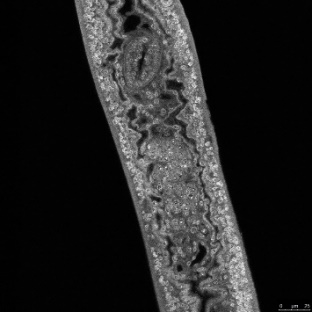


6 d

**unpaired**

**paired**


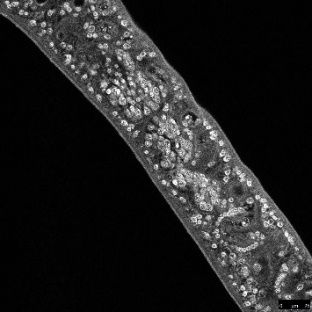

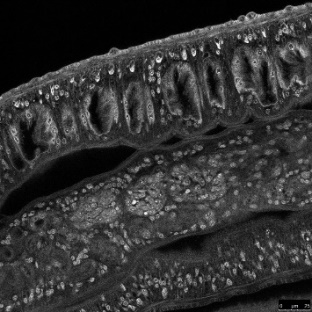

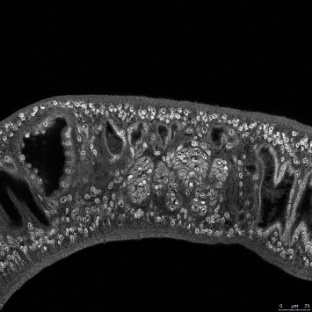

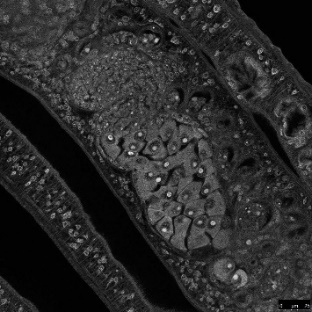


17 d


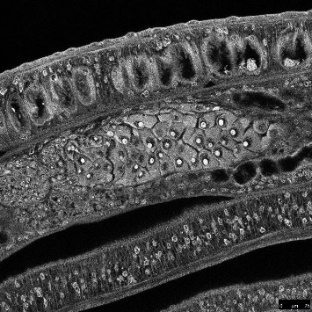

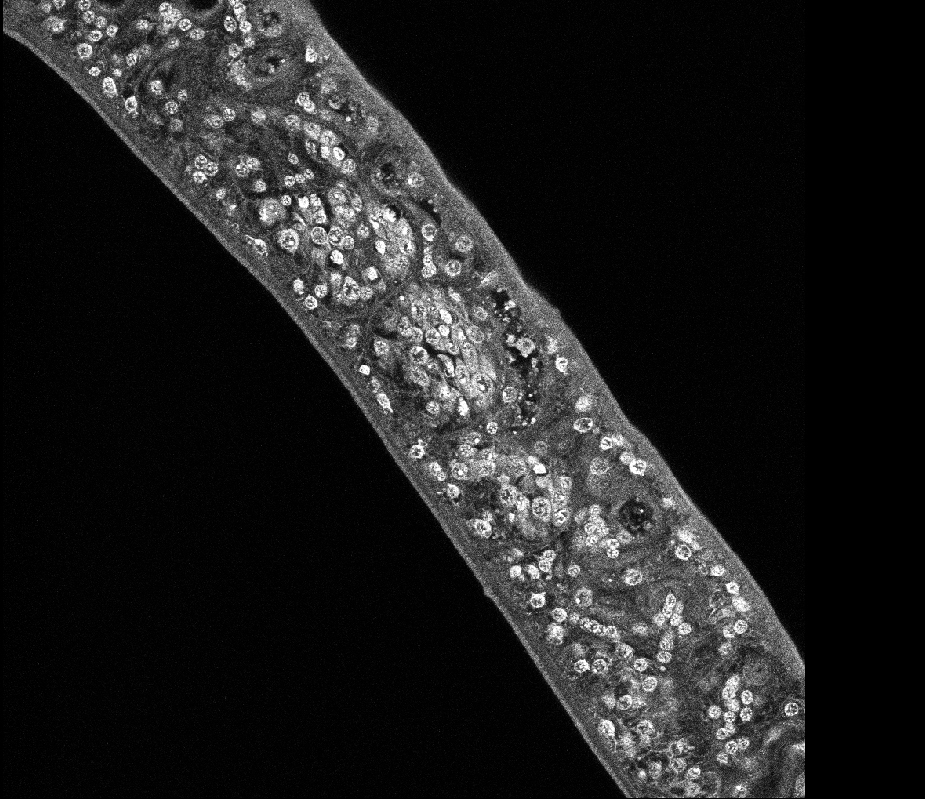


io

io

io

io

io

io

io

mo

io

mo

Suppl. Fig. S2: Under the *in vitro*-culture conditions described above (ABC169/LDL(0,25%)), the time-period for oocyte differentiation was studied in single-sex females upon paring *in vitro*. Following re-pairing with males (male/female ratio of 15:10), single-sex females showed oocyte differentiation within 11 days (d) of repairing. By CLSM, we observed mature oocytes (mo) at d11 and d17 of the experiment. No mature but only immature oocytes (io) were observed in unpaired females kept for the same time-period (d3, d6, d11, and d17) without males in culture. Scale bars: 25 µm.

**Supplementary Figure S3:**

**RNA seq-based transcript profiles of Sm*ddc*-1 and Sm*ddc*-2 in adults and** **gonads**


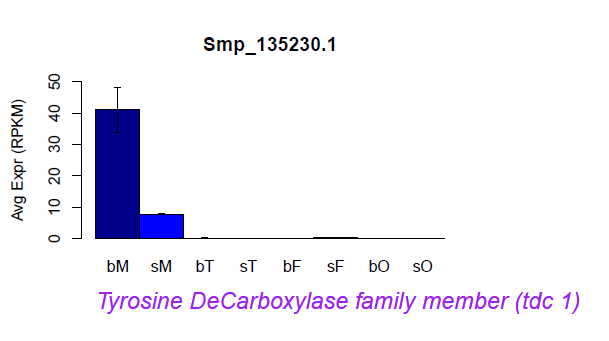

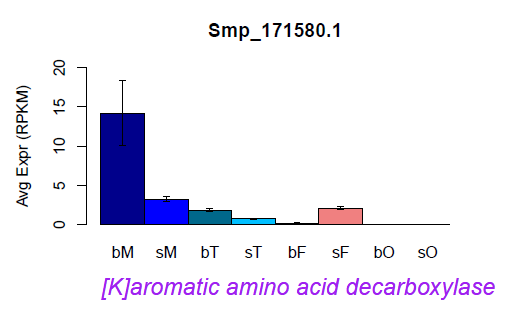


Sm*ddc*-1

Sm*tdc*-1

S

Suppl. Fig. S3: Transcript profiles of Sm*tdc*-1 (Smp_135230, left) and Sm*ddc*-1 (Smp_171580, right) obtained by RNA-seq analysis of female and male *S. mansoni* and their gonads (Lu et al. 2016; Lu et al. 2017). bM, bisex males (pairing-experienced); sM, single-sex males (pairing-unexperienced); bT, testes of bM; sT, testes of sM; bF, bisex females (pairing-experienced); sF, single-sex females (pairing-unexperienced); bO, ovaries from bF; sO, ovaries from sF. Average expression (Avg Expr) was based on RPKM (Reads Per Kilobase per Million mapped reads) values after filtering (Lu et al. 2016).

**Supplementary Figure S4:**

**Structural (A), phylogenetic (B) and alignment analyses (C, D) of Smp_135230 (Sm*tdc*-1) and Smp_171580 (Sm*ddc*-1)**

**A**. **Structural features of in the aligned sequences of Smp_135230 and Smp_171580**

(Motif 1)**FRRRGKEMVDYIADYLDGIE** **VYPDVQPGYLRPLIPATA** (Motif 2)

Smp_135230 MDSHDFNYWGRQMIDFISNYLQTIHKYPVLPNVEPGYLKHLIPNQPPEQSDTWTNIFDDV

Smp_171580 ------------MIQYVADYLENIDERRVFPEVHPGYLAKLLPNEAPNEPESWEEIMNDV

**:::::**:.*.: *:*:*.**** :*:**:.*::.::* :*::**

**PQEPDTFEDILQDV**…

**SPYFFAYFPTASSYPAMLAD** (Motif 4)

Smp_135230 KKFILPSLTHWQHPQFHAYFPAANSVPSIMADMLSTALGCNGFSWVASPAITELEILMCD

Smp_171580 ENMIMPGVTHWQHPHFHAYFPCGCSYTSICADILADGISSIGFTWVSNPACTELELVMID

:::*:*.:******:******.. * .** **:*: .:.. **:**:.** ****::* *

…**EKIIMP** (Motif 3) LCGAIGCIGFSWAASPACT (Motif 5)

(Motif 7)**GGGVIQGSASEATLVALLAAR** **ELETVMMD**…

Smp_135230 WIGKLLNLPETFLHSSGIGGGVIQSSASDCIFVSMLAARHQAIER-YKHLLDMISDL---

Smp_171580 WVAKILSLPEHFLFGEN-SGGVIQGSCSESTLVALLAARNKAIRQYQSI--------HPN

*:.*:*.*** **.... .*****.*.*:. :*::****::**.: .

…**WLGKMLELPEAF** (Motif 6)

Smp_135230 DPEIMVLSRLVAYASK-LAHSAVEKASVLGFVKLRHLPVDENFSIQGETLQRAIKEDKAM

Smp_171580 ASTYEALSKLVGYYSDQ-AHSSVERAGLIGMLHLRAIKSNERYEMNTSILKQTIEDDVNN

. .**:**.* *. ***:**:*.::*:::** : :*.:.:: . *:::*::*

# # # # # #

Smp_135230 GLIPFYVC-ATLGTTSCCSFDHLKSIGQVCRENDIWLHVDAAYAGNAFICPEFRHYLEGI

Smp_171580 GLFPFF-CCATLGTTSTCGFDKLKDIGPICDKYNIWLHIDAAYAGSSFICPEYRYLMDGI

**:**: * ******* *.**:**.** :* : :****:******.:*****:*: ::**

# # **PL**…

Smp_135230 EDAWSININPNKWMLVSHDCSLMWVRDSKALTKSMIVNPSYLQHKYNTL--DFRHWGIPL

Smp_171580 EYAMSFVFNPHKWLLINFDCSIVWYREVNWVKNSFHVDPPYLKHKHQQTTIDFRHMQIPL

* * *: :**:**:*:..***::* *: : :.:*: *:*.**:**:: **** ***

# #

…**GRRFRSLKMWFVFR** (Motif 8) **DPRFEICTEVILGLVCFRL**…

Smp_135230 SRRFRALKLWFVIRIYGATGLRNYIRSHVQLARYFVNKVRANNAYEIVGNPVMGLVCFRL

Smp_171580 GRKFRSLKLWFTLRRYGVKNLQAYIRNHIELAHYFEELIRADDRFEIVAEVLMGLVCFRI

.*:**:*****.:* **...*: ***.*::**:** : :**:: :***.: :*******:

…**K** (Motif 9)

Smp_135230 KGSNELTQCLVHLINTNREIHIVPSMAR--D--IYFIRFSINHEKACIEDIDYSWSVIEK

Smp_171580 KDNNELTKELYHNIEADGRIHLVSSELHLP-KPLYFIRFAICYHSPNKHHIDYAYYVISE

*..****: * * *::: .**:*.* : :*****:* :... ..***:: **.:

Smp_135230 TSRKLLTTQ----HFYKQHLSIMNMKRNSYIPIKAASFSDDHTSSNSTKLF----QRR--

Smp_171580 LCKKLL-LQSNLI--------------------------------------KNKQCNHED

.:*** * .:

Smp_135230 HG----------STSAEESVKVKW

Smp_171580 INLMLLNNENNN------------

Suppl. Fig. S4, **A**: the alignment of Smp_135230 and Smp_171580 showed overall homology of both sequences with 44% identity at the amino acid (aa) level (54% identity at the cDNA level, not shown). According to SMART analysis (Letunic and Bork 2020), pyridoxal domains exist in both sequences (Smp_135230: 35-413, 1.54e^-161^; Smp_171580: positions 23-398, 1.20e^-175^). The start site of these domains is highlighted in yellow. SPRINT analysis exhibited the presence of 9 aa motifs that are typical for aromatic amino acid decarboxylases (<http://130.88.97.239/dbbrowser/sprint/>; Attwood et al. 2003). These motifs are indicated as bold-printed extra sequences above and below the alignment. AA of these motifs that are identical in both genes, Smp_135230 and Smp_171580, are given in red; motif aa occurring in one of both genes are given in blue. There are 10 completely conserved aa found among all group II decarboxylases (Ishii et al. 1996), which are marked with grey background and #. Pink background points to aa that represent the substrate selectivity residues for tyrosine decarboxylases (S) and DOPA decarboxylases (G).

**B. Sequences and accession numbers used for the phylogenetic analyses of Smp_135230 (Sm*tdc*-1), and Smp_171580 (Sm*ddc*-1) in Figure 2**

Genes that belong to one of the different aromatic amino acid decarboxylase groups: tyrosine (TDC), DOPA (DDC), histidine (HDC), glutamate (GDC) decarboxylases, or plant tryptophan decarboxylase (TrpDC). Species include: Sm, *Schistosoma mansoni*; Mm, *Mus musculus* (mouse); Dm, *Drosophila melanogaster* (fly); Cg, *Crassostrea gigas* (oyster); Pc, *Pomacea canaliculata* (snail); *Lymnaea stagnalis* (snail); Cs, *Caenorhabditis elegans* (nematode); Cr, *Catharanthus roseus* (periwinkle plant).

>Smp_135230

MDSHDFNYWGRQMIDFISNYLQTIHKYPVLPNVEPGYLKHLIPNQPPEQSDTWTNIFDDVKKFILPSLTHWQHPQFHAYFPAANSVPSIMADMLSTALGCNGFSWVASPAITELEILMCDWIGKLLNLPETFLHSSGIGGGVIQSSASDCIFVSMLAARHQAIERYKHLLDMISDLDPEIMVLSRLVAYASKLAHSAVEKASVLGFVKLRHLPVDENFSIQGETLQRAIKEDKAMGLIPFYVCATLGTTSCCSFDHLKSIGQVCRENDIWLHVDAAYAGNAFICPEFRHYLEGIEDAWSININPNKWMLVSHDCSLMWVRDSKALTKSMIVNPSYLQHKYNTLDFRHWGIPLSRRFRALKLWFVIRIYGATGLRNYIRSHVQLARYFVNKVRANNAYEIVGNPVMGLVCFRLKGSNELTQCLVHLINTNREIHIVPSMARDIYFIRFSINHEKACIEDIDYSWSVIEKTSRKLLTTQHFYKQHLSIMNMKRNSYIPIKAASFSDDHTSSNSTKLFQRRHGSTSAEESVKVKW

>Smp_171580

MIQYVADYLENIDERRVFPEVHPGYLAKLLPNEAPNEPESWEEIMNDVENMIMPGVTHWQHPHFHAYFPCGCSYTSICADILADGISSIGFTWVSNPACTELELVMIDWVAKILSLPEHFLFGENSGGVIQGSCSESTLVALLAARNKAIRQYQSIHPNASTYEALSKLVGYYSDQAHSSVERAGLIGMLHLRAIKSNERYEMNTSILKQTIEDDVNNGLFPFFCCATLGTTSTCGFDKLKDIGPICDKYNIWLHIDAAYAGSSFICPEYRYLMDGIEYAMSFVFNPHKWLLINFDCSIVWYREVNWVKNSFHVDPPYLKHKHQQTTIDFRHMQIPLGRKFRSLKLWFTLRRYGVKNLQAYIRNHIELAHYFEELIRADDRFEIVAEVLMGLVCFRIKDNNELTKELYHNIEADGRIHLVSSELHLPKPLYFIRFAICYHSPNKHHIDYAYYVISELCKKLLLQSNLIKNKQCNHEDINLMLLNNENNN

>Pc_TDC_XP_025082379.1

MDSLEFRKRGREMIDYIATYMETLSTRRVTPEVEPGYLRHLLPAYPPKKGESFDKIMKDVESAIMPGITHWQHPSFHAYFPAGNSFPSILGDMLSDVIGCIGFSWAASPACTELETLVCDWVGKMIGLPXMFLHESGEGGGVIQGSASECVLVTLLAARHAAIKRLKVRLPFVEDGTLLSRLVAYSSKVAHSCVEKAGFIGFVKMRQLEVDDDFSLRGHVLENAIEEDRRLGLIPFYVCATLGTTACCSFDNIDELGQVCARENLWLHVDAAYAGNALICPEYQHLIHGVENCNSLNFNANKWLQVNFDCSLMWVRNVETLTSALTVDPLYLQHKHGNHTIDLRHWGIPLSRRFRALKLWFVIRTYGVEGLQTIIRQQTILAKTFEEKVLGDDRFEVLGNVTMGLVCFRLQGPNSLSIKLLKMINESGKLHMVPALLNELYVIRFAICSQSASEADVEYAWDVISAFASELLAGRRESATNSDRLSKESASESDDEVFNTDFDDEFIFDHQRCHLQRAHLKRNFFFKMVSDPKSYNPRVLRSLSGRHRSHSVGSSSPGSGEGAYIGQKPVANGAMSNGTPP

>Dm_TDC_NP_724489.1

MDSTEFRKRGMEMVEYICNYLETLNERRVTPSVEPGYLRHLLPPEAPQEPEDWDQIMRDVEDKIMPGVTHWQHPRFHAYFPAGNSFPSILGDMLGDGIGCIGFSWAASPACTELETIVLDWLGKAIGLPDHFLALKEGSTGGGVIQTSASECVLVTMLAARAQALKRLKAQHPFVEEGHLLSKLMAYCSKEAHSCVEKAAMICFVKLRILEPDDDASLRGQTIYEAMEEDELQGLVPFFVSTTLGTTGSCAFDNLPEIGKQLQRFPGVWLHVDAAYAGNSFICPELKPLLKGIEYADSFNTNPNKWLLTNFDCSTLWVRDRIRLTSALVVDPLYLKHGYSDAAIDYRHWGVPLSRRFRSLKLWFVLRSYGISGLQHYIRHHIKLAKRFEELVLKDKRFEICNQVKLGLVCFRLKGSDKLNEKLLSIINESGKLHMVPASVGDRYIIRFCAVAQNATAEDIDYAWDIIVDFANELLEKEQHDELSEIMNRKKQDTLAQKRSFFVRMVSDPKIYNPAINKAGTPKLSMELPSPVVSRGSAPIIRTQSSVDHNSWISWPLAFLFNSNNEEKGSNVSLRFRHLDTNVRPSSSRRNSGAGSSPSPENELDYVNVQQQQMEQRSPRRSPMAVRKASSTRDNLN

>Ce_TDC_NP_495743.1

MVYGLGEALKNLNSYCQERTTRIRNSLSPSRPSMSEATATGSSSSSRASTTIPSTPNMDVTPTVEDPRQNDNNASGMTRDEFRQYGKETVDYIVDYLENIQKRRVVPAIEPGYLKDLIPSEAPNTPESFESVMEDFEKLIMPGITHWQHPRFHAYFPAGNSFPSIIADMLSDAIGCVGFSWAACPAMTELELIMLDWFGKMIGLPAEFLPLTENGKGGGVIQSSASECNFVTLLAARFEVMKELRQRFPFVEEGLLLSKLIAYCSKEAHSSVEKACMIGMVKLRILETDSKFRLRGDTLRNAIQEDRNLGLIPFFVSTTLGTTSCCSFDVLSEIGPICKENELWLHVDAAYSGSAFICPEFRPLMNGIEYAMSFNTNPNKWLLINFDCSTMWVRDRFKLTQALVVDPLYLQHSWMDKSIDYRHWGIPLSRRFRSLKLWFVIRMYGIDGLQKYIREHVRLAKKMETLLRADAKFEIVNEVIMGLVCFRMKGDDELNQTLLTRLNASGRIHMVPASLGDRFVIRFCVCAENATDKDIEVAYEIIAQATQHVLHDSVKAVIAEEDEEAVALEEMVADLNITETPEKCLTRQNSANAAESGQKLERQLSKEEILAQKQHESLAKKRSFLVRMVSDPKCYNPKIVRHLNMANHRKMSQDLYRDRTLMQTISHSQRPNRLSQSPGSAGSAFFDDDDDRIVADVQTGLQTPI

>Cg_TDC_XP_011449820.2

MGDMVKVRKKFNGTTEDRGRTTKKANAMDSVEFRKRGKEMVDFIANYMDTIAERRVTAEVDPGYLRNRLPGKPPRTGDKFEDIMNDVERAIMPGITHWQHPNFHAYFPAGNSYPSILGDMLSNAIGCVGFSWAASPACTELETLVLDWIGKTIGLPKQFLHEEGTGGGVIQGSASECVFVMLLAARHKAMKELKKRLPYIEDGVLLSKLVAYSSKLAHSCVEKAGMLGFVKMRQLDVDVNYSLRGHVLERAIEEDRKLGLIPFFVCGTLGTTACCSFDNVAELSEVCSRENVWLHLDAAYAGNALICPEFRFLINGIQNVTSINFNPNKWLLVNFDCSLLWISDKNLLTSSMTVDPLYLQHKHDDKTVDLRHWGIPLSRRFRALKIWFVLRTYGIEGLQDQIHQHIKLARLFETYVKNDARFEILGKVTMGLVCFRLKGPNSLTAKLLHMINESGKLHMVPALLSEIYVIRFAICSQNARDEDVEFAWKIVSNEASSLLMERENIENGICTEKVGDGDQKNPDIDDVFPDFDDEIIFDQQKSNLHRARLRRSLFMRMVSDPKCYNTKVLKALCVDKKRTRSTPSENSTELDYDK

>Cg_DDC_XP_011417382.2

MDAEEFRRFGKQMVDYVADYLENIRDRKPFPDVSPGYLKELIPDKAPDEAEQWPDVMKDIERVIMPGVTHWHSPQFHAYFPTANSYPAIVADILSDAIGCIGFSWASSPACTELEMVVLDWLAKMLELPDCFLHSSEGHGGGVIQGTASEATLVALLSARTQRLHQILGDKFSHSPDEGIISKMVAYCSAQAHSSVERAALIGAVKVRLLETDEKFSLRGETLQRAIEKDREAGLIPFFLCATLGTTSVCSFDNVLELGTVCEKEGLWMHIDAAYAGSAFICPEFRPLLNGVEHAMSFNFNPHKWLQVNFDCSAMWVKDSRLLSDAFNVDPLYLKHDNQGAIPDFRHWHIPLGRRFRSLKLWFVLRLFGIKGLQERIRKDVKLAHQFEELVKADQRFEIFGEVVLGLVCFRIKGSNEVNERLLKTINDDRRIHLVPSKVNDTFFLRFAVCASRTESKDVKFAWEVIQELTKKISDEKK

>Mm_DDC_NP_001177377.1

MDSREFRRRGKEMVDYIADYLDGIEGRPVYPDVEPGYLRPLIPATAPQEPETYEDIIKDIEKIIMPGVTHWHSPYFFAYFPTASSYPAMLADMLCGAIGCIGFSWAASPACTELETVMMDWLGKMLELPEAFLAGRAGEGGGVIQGSASEATLVALLAARTKVIRQLQAASPEFTQAAIMEKLVAYTSDQAHSSVERAGLIGGIKLKAVPSDGNFSMRASALREALERDKAAGLIPFFVVATLGTTSCCSFDNLLEVGPICNQEGVWLHIDAAYAGSAFICPEFRYLLNGVEFADSFNFNPHKWLLVNFDCSAMWVKRRTDLTGAFNMDPVYLKHSHQDSGFITDYRHWQIPLGRRFRSLKMWFVFRMYGVKGLQAYIRKHVELSHEFESLVRQDPRFEICTEVILGLVCFRLKGSNELNETLLQRINSAKKIHLVPCRLRDKFVLRFAVCARTVESAHVQLAWEHISDLASSVLRAEKE

>Ls_DDC_BAM35936.1

MDAQEFRARGREMVDYVADYLETIGTRTPLPSVLPGYLRELIPDEAPLNGESWEEVKKDIDRVIMPGVTHWHSPQFHAYFPTSSSYPAILGDMLSDGIGCIGFTWPASPACTELEVSMMDWLAKMLNLPQEFLFSGGGKGGGVIQGTASEATLVALLSARTTMINKLKKDNPQMTQGQIVDKLVAYCSEEAHSSVVRASLIGMVQMKSLPTDDKGSLRGSELESAIIKDKEQGLIPFFLCATVGTTSTCGTDNLLELGPICNKHDIWMHVDAAYAGSAFICPEFRPLLDGVEHSMSFNFNPHKWLQVTFDCSALWVKDSGLVSGAFELNPVYLNHDNQGQAMPDYRHWQIPLGRRFRSLKLWFVLRMFGVTGLQEQIRKDVSLAHQFEDLVKSDDRFEIVRKVTFGLVCFRLKGTNEINETLTKKINDDRRIHLTPSKVKDTFFLRFAVCATKTQVSDVKFAWTVIQELTDSLLSSPK

>Dm_DDC_AAO16835.1

MSHIPISNTIPTKQTDGNGKANISPDKLDPKVSIDMEAPEFKDFAKTMVDFIAEYLENIRERRVLPEVKPGYLKPLIPDAAPEKPEKWQDVMQDIERVIMPGVTHWHSPKFHAYFPTANSYPAIVADMLSGAIACIGFTWIASPACTELEVVMMDWLGKMLELPAEFLACSGGKGGGVIQGTASESTLVALLGAKAKKLKEVKELHPEWDEHTILGKLVGYCSDQAHSSVERAGLLGGVKLRSVQSENHRMRGAALEKAIEQDMAEGLIPFYAVVTLGTTNSCAFDYLDECGPVGNKHNLWIHVDAAYAGSAFICPEYRHLMKGIESADSFNFNPHKWMLVNFDCSAMWLKDPSWVVNAFNVDPLYLKHDMQGSAPDYRHWQIPLGRRFRALKLWFVLRLYGVENLQAHIRRHCNFAKQFGDLCVADSRFELAAEINMGLVCFRLKGSNERNEALLKRINGRGHIHLVPAKIKDVYFLRMAICSRFTQSEDMEYSWKEVSAAADEMEQEQ

>Mm_HDC_AAH52833.1

MMEPCEYREYREYYRARGKEMVDYISQYLSTVRERQVTPNVQPGYLRAQLPASAPEEPDSWDSIFGDIERVIMPGVVHWQSPHMHAYYPALTSWPSLLGDMLADAINCLGFTWASSPACTELEMNIMDWLAKMLGLPEYFLHHHPSSRGGGVLQSTVSESTLIALLAARKNKILAMKACEPDANESSLNARLVAYTSDQAHSSVEKAGLISLVKIRFLPVDDNFSLRGEALQKAIEEDKQQGLVPVFVCATLGTTGVCAFDRPSELGPICASEGLWLHVDAAYAGTAFLCPELRGFLEGIEYADSSTFNPSKWMMVHFDCTGFWVKDKYKLQQTFSVNPIYLRHANSGAATDFMHWQIPLSRCFRSIKLWFVIRSFGVKNLQAHVRHGTEMAKYFESLVRSDPSFEIPAKRHLGLVVFRLKGPNCLTESVLKEIAKAGQLFLIPATIQDKLIIRFTVTSQFTTKEDILRDWHLIQEAANLVLSQHCTSQPSPRAKNVIPPPPGTRGLSLESVSEGGDDPAQARKIIKQPGASLARREGGSDLETMPDPFDDCFSEEAPNTTKHKLSSFLFSYLSVQNRRKTTRSLSCNSVPMSAQKSLPADASLKNGGSFRARIFSGFPEQMMMMKKGAFKKLIKFYSVPSFPECSSQCARQLPCCPLEAMV

>Cg_HDC_XP_034320494.1

MDFEEYRLRGKEMVDFIAEYLKSIRTRRVFPDVSPGYMRTLVPEAAPQEGEKWDDIFRDIERVIMPGVTHWQSPYMHAYFPALNSFPSLLGDMLADAIGCLGFTWASSPACTELETIVMDWLGKMIGLPSEFLHSNKETKGGGVIQLTASDCTFITMLAARTEVFQKHRKLDPDVDEAHINARLVAYCSDQAHSSVEKAGLISLVKMRYLTTDDDLSMRGHTLQEAISRDKEDGLIPFYVCATLGTTGACAFDNIKELGLICEKEGLWMHIDAAYAGTAFICPEYRSYIEGIEHANSFAFNPSKWMMVHFDCSAMWVKDCRTLHRTFNVDPLYLKHENSGAAIDYMHWQIPLSRRFRALKLWFVIRSFGIEGLQKHVREGVRLTSKFEDLLRKDQRFEIPASRVLGMVVFRLWGENEMTETLLKRLNKSGKVHMVPASLKGKYVIRFTVTSQYTTDQDIERDWKIISDTATKVLHDTESEEDEAYSDEEVSSPEVEEEKQFVRVPSIKKKEYGMSLLLSNVPMSPKLINGSFAALFDDSEAMEEVAKQISAENAEHLIPMSPRKRKLRDQSKHQSFDQTTMADRCLSNSYKHQGSLDSKLDEIVNSSAYLETRMNGMQMDDVFEAEEERPIDDTEDENCPMKCKDDVSYNEGSTQTETGQRRVDRTPNGQTQSMSQRKNPNIRLRIPEVNIPSMRNVCPHCGNRFSFS

>Ls_HDC_AZK16219.1

MEKMTLSCQPGGMTVEMEKMTSSCPPGGMTVEEYRERAKQTVDYIADYLQTIRSRRVFPDVQPGYMQALVPDAAPVGPDKWEDIFDDVERVIMPGVTHWQSPYMHAYFPALNSYPSLLGDMLANAISSLGFTWASSPACTELETIVMDWLGKMIGLPLHFLHGNKNSKGMGGGCIQTTASDCTFVTLLAARTEAIRQYKSTHPEIDDAEINGRLIGYCSDQAHSSVEKAGLIGLVKMRFLHSDENLSLRGEPLQEAVRMDREKGFIPFYVCATLGTTGACAFDNLKEIGPICKAESLWLHVDAAYAGSAFICPEYRQWMAGIEMVDSFAFNPSKWLMVHFDCSAMWVRDARALHRTFNVEPLYLQHENSGAAIDYMHWQIALSRRFRSLKLWFVIRSFGVEGLQRHIRWGVELAKKFEQLVTSDSRFEVAAKRILGMVVFRIAGANELTEDLLKRLNKQGLVHMVPASLKGKYVIRFTVTSQFTTEEDIERDWRVITDMAKLVLEEGLPQEEESIDEKDEDDENGEGKMVTSMEPFSETMQENGSGLQKDVVVSLNGDTVVKKHPGQHLTSLPKPICVKRRDYGISLLLSNVPMSPKVVNGSYAALFDGGHAGLEQLARQLTVGGEFIRLSPRKRGRLGDFDKQMSLDYSVLGNRRDNPFRMKMMGSLDSKIDDILDLGSKATQTERENIKADTADWKDGTSNVNEVKMAFSNEGRILEQEHWDTGHENWGEGAINGAGSATSGKCKIETVRWDKDMNGKVNTKIKEFDDVKLKYIDGEGSARAKPGRSVVEISKAVCSKVKDVFFTFPGGSPTTTPSKKVNHLSTVALGAPTEHGEKLYCKYCGHVLGSQANGRPGQPTAVKAED

>Dm_HDC_CAA49989.1

MDFKEYRQRGKEMVDYIADYLENIRERRVFPDVSPGYMRQLLPESAPIEGEPWPKIFSDVERIVMPGITHWQSPHMHAYFPALNSMPSLLGDMLADAINCLGFTWASSPACTELEIIVMNWLGKMIGLPDAFLHLSSQSQGGGVLQTTASEATLVCLLAGRTRAIQRFHERHPGYQDAEINARLVAYCSDQAHSSVEKAALIGLVRMRYIEADEDLAMRGKLLREAIEDDIKQGLVPFWVCATLGTTGSCSFDNLEEIGIVCAEHHLWLHVDAAYAGSAFICPEFRTWLRGIERADSIAFNPSKWLMVHFDATALWVRDSTAVHRTFNVEPLYLQHENSGVAVDFMHWQIPLSRRFRALKVWFVLRSYGIKGLQRHIREGVRLAQKFEALVLADHRFELPAKRHLGLVVFRIRGDNEITEKLLKRLNHRGNLHCIPSSLKGQYVIRFTITSTHTTLDDIVKDWMEIRQVASTVLEEMNITISNRVYLKETKEKNEAFGSSLLLSNSPLSPKVVNGSFAAIFDADEFLAKTYAGVRIAHQESPSMRRRVRGILMSGKQFSLDSHMDVVVQTTLDAGNGATRTSTTNSYGHTTSAAQANSERQASIQEDNEESPEETELLSLCRTSNVPSPEHAHSLSTPSRSCSSSSHSLTHSLTQSSARSSPVNQFRHITLCAVPSQSHLSMPLAMPLPNRNVTVSVDSLLNPVTTCNVYHGKRFLEPLENLAQTSASFSSSIFRLPTPMATPTRESPEDPDWPAKTFSQLLLERYSSQSQSLGNNSSTESSSLSGGATPTPTPMSSLDELVTPLLLSFASPSQPMLSAHGIGEGQRERGSDSDATVCSTTSSMESL

>Mm_GDC_NP_032104.2

MASPGSGFWSFGSEDGSADPENPGTARAWCQVAQKFTGGIGNKLCALLYGDSGKPAEGGGSVTSRAATGKVACTCDQKPCNCPKGDVNYAFLHATDLLPACDGERPTLAFLQDVMNILLQYVVKSFDRSTKVIDFHYPNELLQEYNWELADQPQNLEEILTHCQTTLKYAIKTGHPRYFNQLSTGLDMVGLAADWLTSTANTNMFTYEIAPVFVLLEYVTLKKMREIIGWPGGSGDGIFSPGGAISNMYAMLIARYKMFPEVKEKGMAAVPRLIAFTSEHSHFSLKKGAAALGIGTDSVILIKCDERGKMIPSDLERRILEVKQKGFVPFLVSATAGTTVYGAFDPLLAVADICKKYKIWMHVDAAWGGGLLMSRKHKWKLSGVERANSVTWNPHKMMGVPLQCSALLVREEGLMQSCNQMHASYLFQQDKHYDLSYDTGDKALQCGRHVDVFKLWLMWRAKGTTGFEAHIDKCLELAEYLYTIIKNREGYEMVFDGKPQHTNVCFWFVPPSLRTLEDNEERMSRLSKVAPVIKARMMEYGTTMVSYQPLGDKVNFFRMVISNPAATHQDIDFLIEEIERLGQDL

>Cg_GDC_XP_034310760.1

MKSIGTSLARAALRKLKIDLAKISQPRLFSSMASKDREDAFSNPDVQQMLLNLHHSMVKTLIDSETKELPVCNFKQPEELLKALDLEIEKEPASNDTILDACKSVMDYSIKTASPRFMNQLYSGINPSCLAGSWVTEVLNTNLHTYEVAPVFVMMEKYMMRKLSKLVGYDNGDGVLCPGGSFANMLGMHLARVQIDPDIKSKGMGNNKKMVLFASSEAHYSIAKGASFLGFGESNVVRVETDKIGMMKPDVLDQKIQECIQQGSIPAFVMATTGSTVLGSCDDLNAVADVCEKHGVWMHVDAAWGGGVILSEKYKHLMNGVHRSNSVAWNIHKMSTGLVQCSIFLTKSSGMMEECNRFNAEYLFQPDKHYDVSYDIGDKTVQCGRKVDILKLWTLWKSRGDNGMAKQTDNAFENAKYLVDQIKKREGFRLVLPEFQCPNICFWYIPSRLRNKEENADWWKEVSKIAPQIKRKIMEDGSMMIGYNPLTTKGYVNFFRVIITNPMTTPQDMDFILDEMDRIGHAL

>Dm_GDC_CAA53791.1

MSLNPNGYKLSERTGKLTAYDLMPTTVTAGPETREFLLKVIDVLLDFVKATNDRNEKVLDFHHPEDMKRLLDLDVPDRALPLQQLIEDCATTLKYQVKTGHPHFFNQLSNGLDLISMAGEWLTATANTNMFTYEIAPVFILMENVVLTKMREIIGWSGGDSILAPGGSISNLYAFLAARHKMFPNYKEHGSVGLPGTLVMLTSDQCHYSIKSCAAVCGLGTDHCIVVPSDEHGKMITSELERLILERKAKGDIPFFVNATAGTTVLGAFDDINTIADICQKYNCWMHIDAAWGGGLLMSRTHRHPRFTGVERADSVTWNPHKLMGALLQCSTIHFKEDGLLISCNQMSAEYLFMTDKQYDISYDTGDKVIQCGRHNDIFKLWLQWRAKGTEGFEQQQDRLMELVQYQLKRIREQSDRFHLILEPECVNVSFWYVPKRLRGVPHDAKKEVELGKICPIIKGRMMQKGTLMVGYQPDDRRPNFFRSIISSAAVNEADVDFMLDEIHRLGDDL

>Pc_GDC_XP_025080415.1

MDWAQMKTAETIGVGSRVNSPMLTEHSQCDSNKEVDSQEAEGQMAYQCGMFLNKLKRGIVNHTSSHGSNTASEEGPQQGKDSHAAIICKSSDWSQFEGVLESDFFNAGGEGKACKFLREVALVLMQYYVAEMDRKTKVVDFHHPHQLREMMSHCLDIDETPRDLEQLLSDCKETLKYCVKTGHGHYIQQQLSTGIDVVGVAGEWLTAIANTNMFFLYEVGAVFXLMEIVGAFDLLGENCWENEGEGIFAPGGAVSNLYGVLIARHSVFPKAKEEGMPWGVCPVVFTSEHSHFSIKRACALLGIGTNNCIRVRCDHRGKMLVEELEQHILAAKAAGKTPVMVNAMCGTTGLGAIDPLDAIADVCRTYGIWMHVDGAWGGALLMSRDHRYLMKGIERADSMTWNPHKMMGVPLQCSAILCKRKGILQAANQMKADYLFQQDKHYDLSWDTGDKTIQCGRHNDIFKMWLMWRAKGDEGFERQVVHNMEMAKYLKDRATDHKGFHIVNTELDAPNVCFWYLPEAWRHIPFEQIKKEQITKALMMESGTTMVQYQPLGTMPNFFRVAVSNPILTTQDMDFLVDEIDRLGRDIPYPILDA

>Ce_GDC_AAD19958.1

MSSAAADESDAVLENLIAKEILPQTGNWEGTEEFLNRIVQVLLKYIKDQNDRDQKILEFHHPDKMQMLMDLSIPEKPESLLKLVKSCEDVLRLGVRTGHPRFFNQISCGLDLVSMAGEWLTATANTNMFTYEIAPVFILMEKSVMARMWEAVGWDPEKADGIFAPGGAIANLYAMNAARHQLWPRSKHLGMKDIPTLCCFTSEDSHYSIKSASAVLGIGADYCFNIPTDKNGKMIPEALEAKIIECKKEGLTPFFACCTAGSTVYGAFDPLERVANICERHKLWFHVDAAWGGGMLLSPEHRYKLAGIERANSVTWNPHKLMGALLQCSACLFRQDGLLFQCNQMSADYLFQQDKPYDVSFDTGDKAIQCGRHNDVFKLWLMWKSKGMEGYRQQINKLMDLANYFTRRIKETEGFELIIENPEFLNICFWYVPSKIRNLEPAEMRARLEKIAPKIKAGMMQRGTTMVGYQPDKQRPNFFRMIISNQAITREDLDFLIKEIVDIGESLE

>Cr_TrpDC_CAA47898.1

MGSIDSTNVAMSNSPVGEFKPLEAEEFRKQAHRMVDFIADYYKNVETYPVLSEVEPGYLRKRIPETAPYLPEPLDDIMKDIQKDIIPGMTNWMSPNFYAFFPATVSSAAFLGEMLSTALNSVGFTWVSSPAATELEMIVMDWLAQILKLPKSFMFSGTGGGVIQNTTSESILCTIIAARERALEKLGPDSIGKLVCYGSDQTHTMFPKTCKLAGIYPNNIRLIPTTVETDFGISPQVLRKMVEDDVAAGYVPLFLCATLGTTSTTATDPVDSLSEIANEFGIWIHVDAAYAGSACICPEFRHYLDGIERVDSLSLSPHKWLLAYLDCTCLWVKQPHLLLRALTTNPEYLKNKQSDLDKVVDFKNWQIATGRKFRSLKLWLILRSYGVVNLQSHIRSDVAMGKMFEEWVRSDSRFEIVVPRNFSLVCFRLKPDVSSLHVEEVNKKLLDMLNSTGRVYMTHTIVGGIYMLRLAVGSSLTEEHHVRRVWDLIQKLTDDLLKEA

**C. Multiple alignment of amino acid sequences encoding L-tyrosine decarboxylases (TDCs), including Smp_135230 and Smp_171580**

We used Clustal Omega for the alignment. Depicted species and accession numbers include *Caenorhabditis elegans*, NP_495743.1; *Drosophila melanogaster*, NP_724489.1; *Crassostrea gigas*, XP_011449820.2; *Pomacea canaliculata*, XP_025082379.1; *Schistosoma mansoni*, Smp_135230. The pyridoxal 5′-phosphate-binding residue of group II pyridoxal-dependent DCs is highlighted in red, the conserved histidine residue in the small catalytic loop (within box) characteristic for AADCs is given in yellow, and the substrate selectivity residue (serine) characteristic for TDCs is in pink. 100% identical amino acids are marked by asterisks.

C.elegans MVYGLGEALKNLNSYCQERTTRIRNSLSPSRPSMSEATATGSSSSSRASTTIPSTPNMDV

D.melanogaster ------------------------------------------------------------

C.gigas ---------------------------------------MGDM--------V--KVRKKF

P.canaliculata ------------------------------------------------------------

S.mansoni ------------------------------------------------------------

C.elegans TPTVEDPRQNDNNASGMTRDEFRQYGKETVDYIVDYLENIQKRRVVPAIEPGYLKDLIPS

D.melanogaster ----------------MDSTEFRKRGMEMVEYICNYLETLNERRVTPSVEPGYLRHLLPP

C.gigas NGTTEDRGRTTKKANAMDSVEFRKRGKEMVDFIANYMDTIAERRVTAEVDPGYLRNRLPG

P.canaliculata ----------------MDSLEFRKRGREMIDYIATYMETLSTRRVTPEVEPGYLRHLLPA

S.mansoni ----------------MDSHDFNYWGRQMIDFISNYLQTIHKYPVLPNVEPGYLKHLIPN

* :*. * : :::* *::.: * ::****:. :*

C.elegans EAPNTPESFESVMEDFEKLIMPGITHWQHPRFHAYFPAGNSFPSIIADMLSDAIGCVGFS

D.melanogaster EAPQEPEDWDQIMRDVEDKIMPGVTHWQHPRFHAYFPAGNSFPSILGDMLGDGIGCIGFS

C.gigas KPPRTGDKFEDIMNDVERAIMPGITHWQHPNFHAYFPAGNSYPSILGDMLSNAIGCVGFS

P.canaliculata YPPKKGESFDKIMKDVESAIMPGITHWQHPSFHAYFPAGNSFPSILGDMLSDVIGCIGFS

S.mansoni QPPEQSDTWTNIFDDVKKFILPSLTHWQHPQFHAYFPAANSVPSIMADMLSTALGCNGFS

*. : : .:: *.: *:*.:****** *******.** ***:.***. :** ***

C.elegans WAACPAMTELELIMLDWFGKMIGLPAEFLPLTENGKGGGVIQSSASECNFVTLLAARFEV

D.melanogaster WAASPACTELETIVLDWLGKAIGLPDHFLALKEGSTGGGVIQTSASECVLVTMLAARAQA

C.gigas WAASPACTELETLVLDWIGKTIGLPKQFL--HEEGTGGGVIQGSASECVFVMLLAARHKA

P.canaliculata WAASPACTELETLVCDWVGKMIGLPXMFL--HESGEGGGVIQGSASECVLVTLLAARHAA

S.mansoni WVASPAITELEILMCDWIGKLLNLPETFL--HSSGIGGGVIQSSASDCIFVSMLAARHQA

*.*.** **** :: **.** :.** ** . . ****** ***:* :* :**** .

C.elegans MKELRQRFPFVE----EGLLLSKLIAYCSKEAHSSVEKACMIGMVKLRILETDSKFRLRG

D.melanogaster LKRLKAQHPFVE----EGHLLSKLMAYCSKEAHSCVEKAAMICFVKLRILEPDDDASLRG

C.gigas MKELKKRLPYIE----DGVLLSKLVAYSSKLAHSCVEKAGMLGFVKMRQLDVDVNYSLRG

P.canaliculata IKRLKVRLPFVE----DGTLLSRLVAYSSKVAHSCVEKAGFIGFVKMRQLEVDDDFSLRG

S.mansoni IERYKHLLDMISDLDPEIMVLSRLVAYASKLAHSAVEKASVLGFVKLRHLPVDENFSIQG

::. : :. : :**:*:**.** ***.**** .: :**:* * * . ::*

C.elegans DTLRNAIQEDRNLGLIPFFVSTTLGTTSCCSFDVLSEIGPICK-ENELWLHVDAAYSGSA

D.melanogaster QTIYEAMEEDELQGLVPFFVSTTLGTTGSCAFDNLPEIGKQLQRFPGVWLHVDAAYAGNS

C.gigas HVLERAIEEDRKLGLIPFFVCGTLGTTACCSFDNVAELSEVCS-RENVWLHLDAAYAGNA

P.canaliculata HVLENAIEEDRRLGLIPFYVCATLGTTACCSFDNIDELGQVCA-RENLWLHVDAAYAGNA

S.mansoni ETLQRAIKEDKAMGLIPFYVCATLGTTSCCSFDHLKSIGQVCR-ENDIWLHVDAAYAGNA

..: .*::**. **:**:*. *****..*:** : .:. :***:****:*.:

C.elegans FICPEFRPLMNGIEYAMSFNTNPNKWLLINFDCSTMWVRDRFKLTQALVVDPLYLQHSWM

D.melanogaster FICPELKPLLKGIEYADSFNTNPNKWLLTNFDCSTLWVRDRIRLTSALVVDPLYLKHGYS

C.gigas LICPEFRFLINGIQNVTSINFNPNKWLLVNFDCSLLWISDKNLLTSSMTVDPLYLQHKHD

P.canaliculata LICPEYQHLIHGVENCNSLNFNANKWLQVNFDCSLMWVRNVETLTSALTVDPLYLQHKHG

S.mansoni FICPEFRHYLEGIEDAWSININPNKWMLVSHDCSLMWVRDSKALTKSMIVNPSYLQHKYN

:**** : :.*:: *:* * ***: ..*** :*: : **.:: *:* **:*

C.elegans DKSIDYRHWGIPLSRRFRSLKLWFVIRMYGIDGLQKYIREHVRLAKKMETLLRADAKFEI

D.melanogaster DAAIDYRHWGVPLSRRFRSLKLWFVLRSYGISGLQHYIRHHIKLAKRFEELVLKDKRFEI

C.gigas DKTVDLRHWGIPLSRRFRALKIWFVLRTYGIEGLQDQIHQHIKLARLFETYVKNDARFEI

P.canaliculata NHTIDLRHWGIPLSRRFRALKLWFVIRTYGVEGLQTIIRQQTILAKTFEEKVLGDDRFEV

S.mansoni --TLDFRHWGIPLSRRFRALKLWFVIRIYGATGLRNYIRSHVQLARYFVNKVRANNAYEI

::* ****:*******:**:***:* ** **: *: : **: : : : :*:

C.elegans VNEVIMGLVCFRMKGDDELNQTLLTRLNASGRIHMVPASLGDRFVIRFCVCAENATDKDI

D.melanogaster CNQVKLGLVCFRLKGSDKLNEKLLSIINESGKLHMVPASVGDRYIIRFCAVAQNATAEDI

C.gigas LGKVTMGLVCFRLKGPNSLTAKLLHMINESGKLHMVPALLSEIYVIRFAICSQNARDEDV

P.canaliculata LGNVTMGLVCFRLQGPNSLSIKLLKMINESGKLHMVPALLNELYVIRFAICSQSASEADV

S.mansoni VGNPVMGLVCFRLKGSNELTQCLVHLINTNREIHIVPSMARDIYFIRFSINHEKACIEDI

.: :******::* :.*. *: :* . .:*:**: : :.***. :.* *:

C.elegans EVAYEIIAQATQHVLHDSVKAVIAEE-----DEEAVALEE--MVADLNITETPEKCLTRQ

D.melanogaster DYAWDIIVDFANELLEKEQHDELSE-----------------------------------

C.gigas EFAWKIVSNEASSLLMERENIENGI------------------------------CTEKV

P.canaliculata EYAWDVISAFASELLAGRRESAT--------------------------------NSDRL

S.mansoni DYSWSVIEKTSRKLLTTQHFYKQHLSIMNMKRNSYIPIKAASFSDDHTSSNSTKLFQRRH

: ::.:: : :*

C.elegans NSANAAESGQ-KLERQLSKEEIL--AQKQHESLAKKRSFLVRMVSDPKCYNPKIVRHLNM

D.melanogaster --------------------I---MNRKKQDTLAQKRSFFVRMVSDPKIYNPAINKAGTP

C.gigas GDGDQKNPDIDDVFPDFDDEIIFDQQKSNLHRARLRRSLFMRMVSDPKCYNTKVLKALCV

P.canaliculata SKESASESDDEVFNTDFDDEFIFDHQRCHLQRAHLKRNFFFKMVSDPKSYNPRVLRSLSG

S.mansoni GSTSAEESVKVKW-----------------------------------------------

C.elegans ANHRKMSQDLYRDRTLMQTISHSQRPNRLSQSPGSAGSAFFDDDDDRIVADVQ-------

D.melanogaster KLSMELPSPVVSRGSA--PIIRTQSSVDHNSWISWPLAFLFNSNNEEKGSNVSLRFRHLD

C.gigas DKKRTRSTP--SENSTELDYDK--------------------------------------

P.canaliculata RHRSHSVGSS-SPGSGEGAYIG-QKPVANG------------------------------

S.mansoni ------------------------------------------------------------

C.elegans -----------TGLQ--------------------------TPI-------------

D.melanogaster TNVRPSSSRRNSGAGSSPSPENELDYVNVQQQQMEQRSPRRSPMAVRKASSTRDNLN

C.gigas ---------------------------------------------------------

P.canaliculata ----------AMSNGTPP---------------------------------------

S.mansoni ---------------------------------------------------------

**D. Multiple alignment of amino acid sequences encoding DOPA decarboxylases (DDCs) including Smp_171580**

We used Clustal Omega for the alignment. Depicted species and accession numbers include *Mus musculus*, NP_001177377.1; *Drosophila melanogaster*, AAO16835.1; *Crassostrea gigas*, XP_011417382.2; *Lymnaea stagnalis*, BAM35936.1; *Schistosoma mansoni*, Smp_171580. The pyridoxal 5′-phosphate-binding residue of group II pyridoxal-dependent DCs is highlighted in red, the conserved histidine residue in the small catalytic loop (within box) characteristic for AADCs is given in yellow, and the substrate selectivity residue (glycine) characteristic for DDCs is in pink. 100% identical amino acids are marked by asterisks.

M.musculus -----------------------------------MDSREFRRRGKEMVDYIADYLDGIE

D.melanogaster MSHIPISNTIPTKQTDGNGKANISPDKLDPKVSIDMEAPEFKDFAKTMVDFIAEYLENIR

C.gigas -----------------------------------MDAEEFRRFGKQMVDYVADYLENIR

L.stagnalis -----------------------------------MDAQEFRARGREMVDYVADYLETIG

S.mansoni -----------------------------------------------MIQYVADYLENID

*::::*:**: *

M.musculus GRPVYPDVEPGYLRPLIPATAPQEPETYEDIIKDIEKIIMPGVTHWHSPYFFAYFPTASS

D.melanogaster ERRVLPEVKPGYLKPLIPDAAPEKPEKWQDVMQDIERVIMPGVTHWHSPKFHAYFPTANS

C.gigas DRKPFPDVSPGYLKELIPDKAPDEAEQWPDVMKDIERVIMPGVTHWHSPQFHAYFPTANS

L.stagnalis TRTPLPSVLPGYLRELIPDEAPLNGESWEEVKKDIDRVIMPGVTHWHSPQFHAYFPTSSS

S.mansoni ERRVFPEVHPGYLAKLLPNEAPNEPESWEEIMNDVENMIMPGVTHWQHPHFHAYFPCGCS

* *.* **** *:* ** : * : :: :*::.:********: * *.**** . *

M.musculus YPAMLADMLCGAIGCIGFSWAASPACTELETVMMDWLGKMLELPEAFLAGRAGEGGGVIQ

D.melanogaster YPAIVADMLSGAIACIGFTWIASPACTELEVVMMDWLGKMLELPAEFLACSGGKGGGVIQ

C.gigas YPAIVADILSDAIGCIGFSWASSPACTELEMVVLDWLAKMLELPDCFLHSSEGHGGGVIQ

L.stagnalis YPAILGDMLSDGIGCIGFTWPASPACTELEVSMMDWLAKMLNLPQEFLFSGGGKGGGVIQ

S.mansoni YTSICADILADGISSIGFTWVSNPACTELELVMIDWVAKILSLPEHFLFGE--NSGGVIQ

* :: .*:*...*..***:* :.******* ::**:.*:*.** ** ..*****

M.musculus GSASEATLVALLAARTKVIRQLQAAS-PEFTQAAIMEKLVAYTSDQAHSSVERAGLIGGI

D.melanogaster GTASESTLVALLGAKAKKLKEVKELH-PEWDEHTILGKLVGYCSDQAHSSVERAGLLGGV

C.gigas GTASEATLVALLSARTQRLHQILGDKFSHSPDEGIISKMVAYCSAQAHSSVERAALIGAV

L.stagnalis GTASEATLVALLSARTTMINKLKKDN-PQMTQGQIVDKLVAYCSEEAHSSVVRASLIGMV

S.mansoni GSCSESTLVALLAARNKAIRQYQSIH-PNASTYEALSKLVGYYSDQAHSSVERAGLIGML

*:.**:******.*: :.: . : *:*.* * :***** **.*:* :

M.musculus KLKAVPSDGNFSMRASALREALERDKAAGLIPFFVVATLGTTSCCSFDNLLEVGPICNQE

D.melanogaster KLRSVQSE-NHRMRGAALEKAIEQDMAEGLIPFYAVVTLGTTNSCAFDYLDECGPVGNKH

C.gigas KVRLLETDEKFSLRGETLQRAIEKDREAGLIPFFLCATLGTTSVCSFDNVLELGTVCEKE

L.stagnalis QMKSLPTDDKGSLRGSELESAIIKDKEQGLIPFFLCATVGTTSTCGTDNLLELGPICNKH

S.mansoni HLRAIKSNERYEMNTSILKQTIEDDVNNGLFPFFCCATLGTTSTCGFDKLKDIGPICDKY

::: : :: . :. *. :: * **:**: .*:***. *. * : : * : ::

M.musculus GVWLHIDAAYAGSAFICPEFRYLLNGVEFADSFNFNPHKWLLVNFDCSAMWVKRRTDLTG

D.melanogaster NLWIHVDAAYAGSAFICPEYRHLMKGIESADSFNFNPHKWMLVNFDCSAMWLKDPSWVVN

C.gigas GLWMHIDAAYAGSAFICPEFRPLLNGVEHAMSFNFNPHKWLQVNFDCSAMWVKDSRLLSD

L.stagnalis DIWMHVDAAYAGSAFICPEFRPLLDGVEHSMSFNFNPHKWLQVTFDCSALWVKDSGLVSG

S.mansoni NIWLHIDAAYAGSSFICPEYRYLMDGIEYAMSFVFNPHKWLLINFDCSIVWYREVNWVKN

.:*:*:*******:*****:* *:.*:* : ** ******: :.**** :* : : .

M.musculus AFNMDPVYLKHSHQDSGFITDYRHWQIPLGRRFRSLKMWFVFRMYGVKGLQAYIRKHVEL

D.melanogaster AFNVDPLYLKHDMQG--SAPDYRHWQIPLGRRFRALKLWFVLRLYGVENLQAHIRRHCNF

C.gigas AFNVDPLYLKHDNQG--AIPDFRHWHIPLGRRFRSLKLWFVLRLFGIKGLQERIRKDVKL

L.stagnalis AFELNPVYLNHDNQGQ-AMPDYRHWQIPLGRRFRSLKLWFVLRMFGVTGLQEQIRKDVSL

S.mansoni SFHVDPPYLKHKHQQ--TTIDFRHMQIPLGRKFRSLKLWFTLRRYGVKNLQAYIRNHIEL

:*.::* **:*. * *:** :*****:**:**:**.:* :*: .** **.. .:

M.musculus SHEFESLVRQDPRFEICTEVILGLVCFRLKGSNELNETLLQRINSAKKIHLVPCRL---R

D.melanogaster AKQFGDLCVADSRFELAAEINMGLVCFRLKGSNERNEALLKRINGRGHIHLVPAKI---K

C.gigas AHQFEELVKADQRFEIFGEVVLGLVCFRIKGSNEVNERLLKTINDDRRIHLVPSKV---N

L.stagnalis AHQFEDLVKSDDRFEIVRKVTFGLVCFRLKGTNEINETLTKKINDDRRIHLTPSKV---K

S.mansoni AHYFEELIRADDRFEIVAEVLMGLVCFRIKDNNELTKELYHNIEADGRIHLVSSELHLPK

:: * .* * ***: :: :******:*..** .: * : *: :***. ..: .

M.musculus DKFVLRFAVCARTVESAHVQLAWEHISDLASSVLRAEKE---------------------

D.melanogaster DVYFLRMAICSRFTQSEDMEYSWKEVSAAADEMEQEQ-----------------------

C.gigas DTFFLRFAVCASRTESKDVKFAWEVIQELTKKISDEKK----------------------

L.stagnalis DTFFLRFAVCATKTQVSDVKFAWTVIQELTDSLLSSPK----------------------

S.mansoni PLYFIRFAICYHSPNKHHIDYAYYVISELCKKLLLQSNLIKNKQCNHEDINLMLLNNENN

:.:*:*:* : .:. :: :. ..:

**Supplementary Figure S5:**

**RNA seq-based single-cell atlas of *S. mansoni* (A) with focus on transcript profiles of Sm*tdc-1* (B), and Sm*ddc-1* (C)**

**A. Cell atlas of *S. mansoni***

**
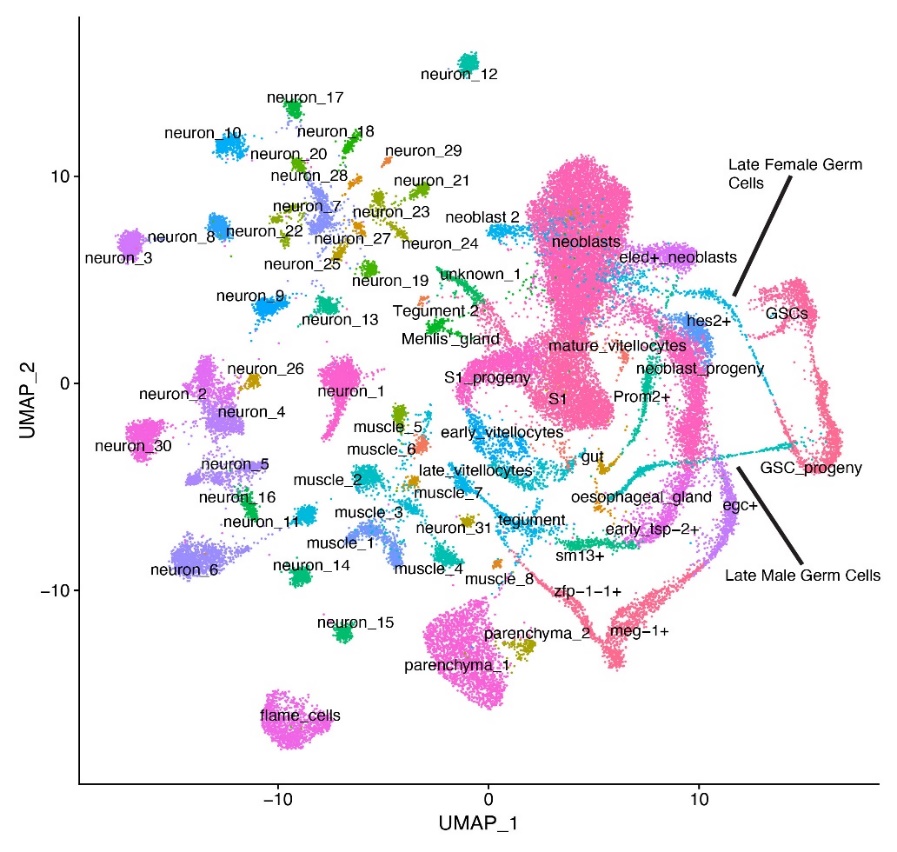
**

Suppl. Fig. S5, **A**: This cell atlas was obtained by single-cell RNAseq analysis (Wendt et al. 2020). The overview shows cell clusters assigned to various tissues of *S. mansoni*. Highlighted is a neuronal cluster (black circle) that includes neuron 2 (arrow) for comparison with data obtained for Sm*tdc-1* and Sm*ddc-1*, see below and manuscript text.

**B. Transcript profile of Sm*tdc-1* within the cell atlas**


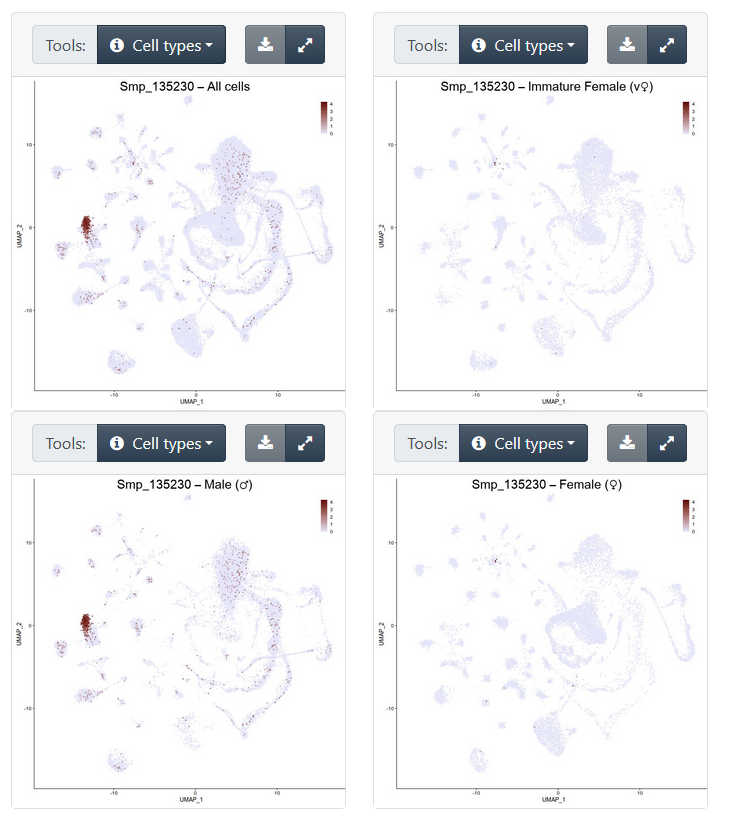


Suppl. Fig. S5, **B**: According to the cell-atlas data, Sm*tdc-1* is preferentially transcribed in neuron 2 cells (All cells, black circle) and weakly also in other neuronal cells, neoblasts, and tegumental cells (Wendt et al. 2020). With respect to sex-preferential expression, the data correspond to RNAseq data of adults obtained in a preceding study (Lu et al. 2016; see Supplementary Figure 2). Note the difference of neuron 2 cells (black circle in Smp_135230 - All cells) between males (bottom left, Male, blue circle) and females (bottom right, Female, red circle) with respect to gene expression and cell-content of this neuronal cluster.

**C. Transcript profile of Sm*ddc-1* within the cell atlas**


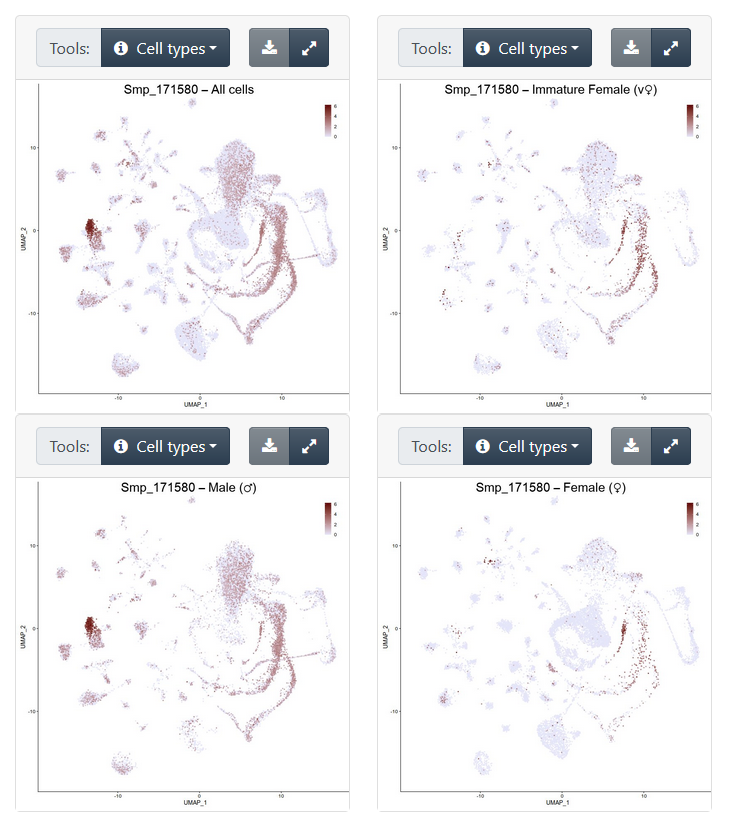


Suppl. Fig. S5, **C**: According to cell-atlas data, Sm*ddc-1* is preferentially expressed in neuron 2 cells (All cells, black circle) and weakly also in other neuronal cells, neoblasts, parenchyma, flame cells, precursor gut cells, and tegumental cells. With respect to sex-preferential expression, the data correspond to the RNAseq data of adults obtained in a preceding study (Lu et al. 2016; see Supplementary Figure 2). Again, note the difference of neuron 2 cells between males (Male, blue circle) and females (Female, red circle) with respect to gene expression and cell-content of this neuronal cluster.

**Supplementary Figure 6:**

**Sm*tdc-1* and Sm*ddc-1* knock-down analyses (A) and its influence on re-pairing (B), egg production (C), and the status of oocyte differentiation (D) in paired females of group 1**

**A. RNAi against Sm*tdc-1* and Sm*ddc-1* significantly reduced transcript levels of both genes**

**
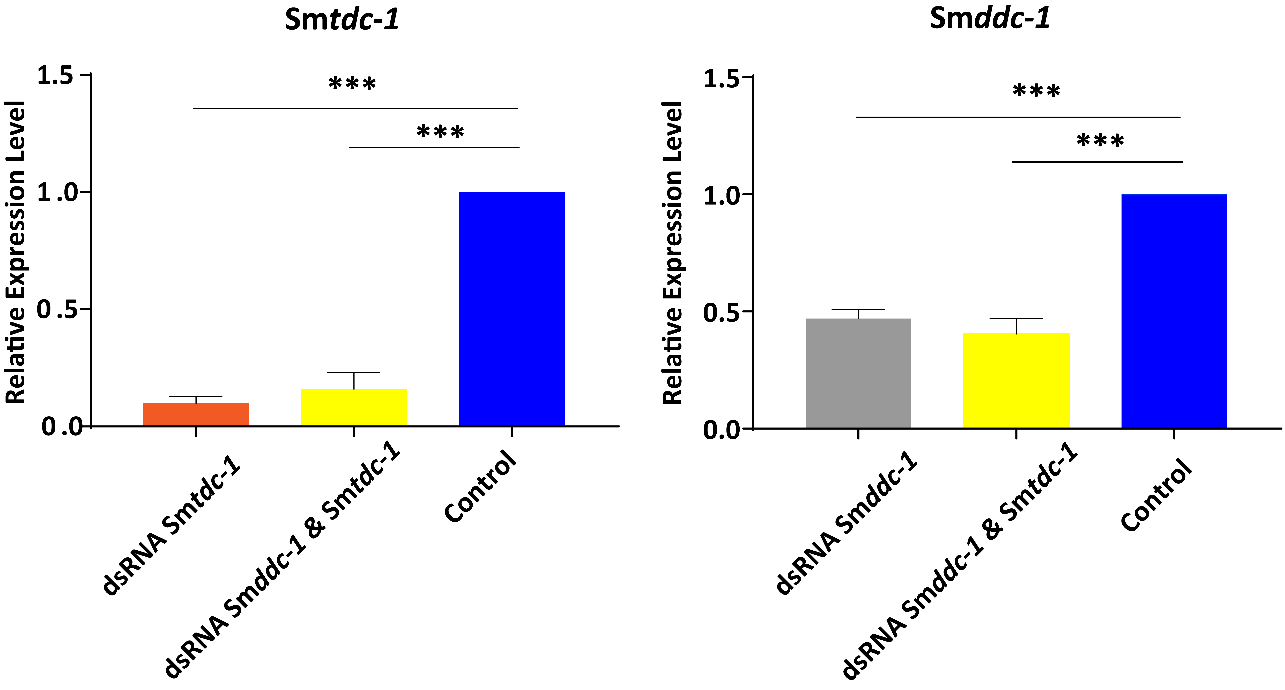
**

= Control = dsRNA against Sm*ddc-1* = dsRNA against Sm*tdc-1*

= dsRNAs against Sm*tdc-1* and Sm*ddc-1*

**B. Effect of RNAi against Sm*tdc-1* and Sm*ddc-1* slightly on repairing and pairing stability**

**
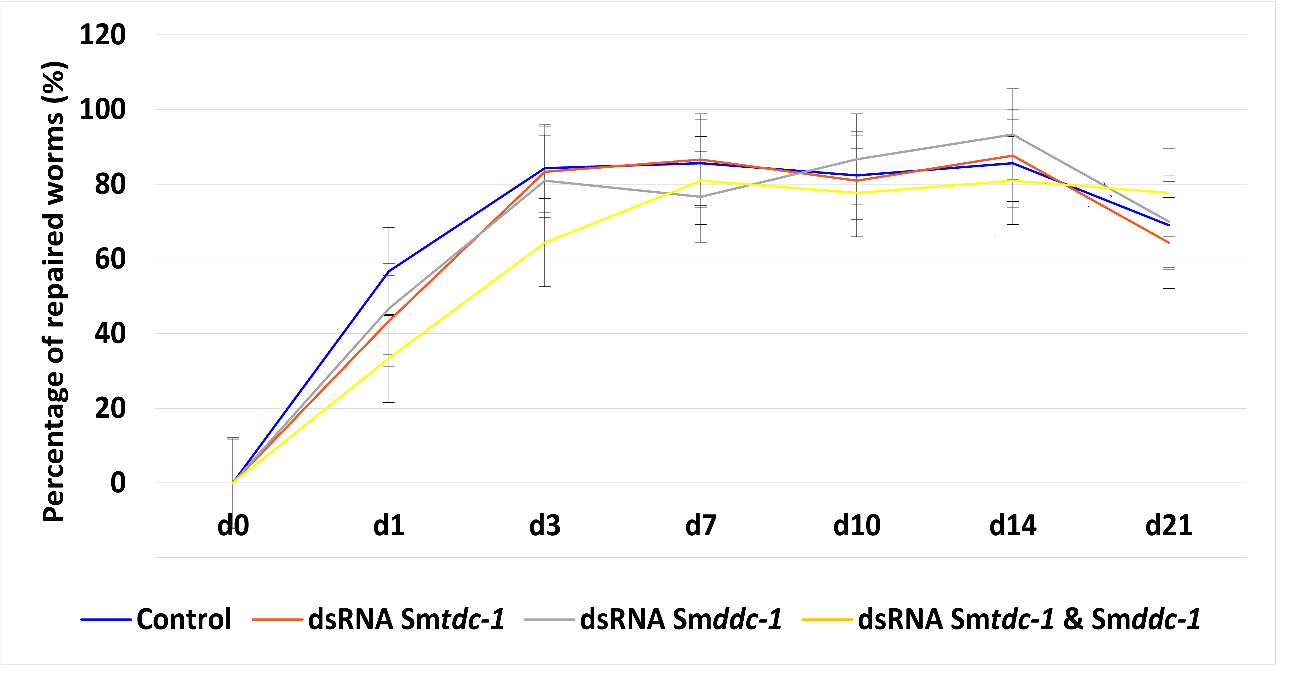
**

**C. RNAi against Sm*tdc-1* and Sm*ddc-1* reduced egg-production rates of paired females**


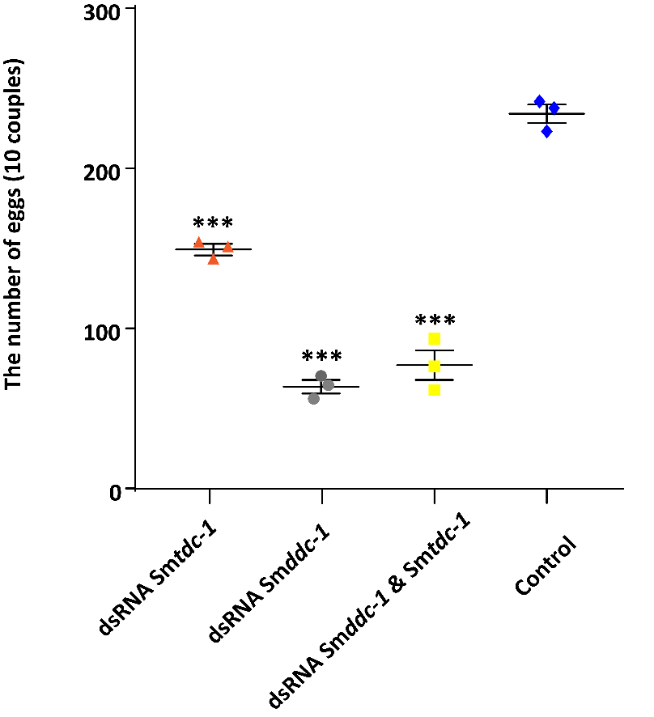


**D. RNAi against Sm*tdc-1* and Sm*ddc-1* reduced the number of differentiated oocytes of paired females**

mo

io

m

m

**Sm*tdc-1***

**Sm*ddc-1***

mo

io

f

m

m

**Control**

mo

io

m

m


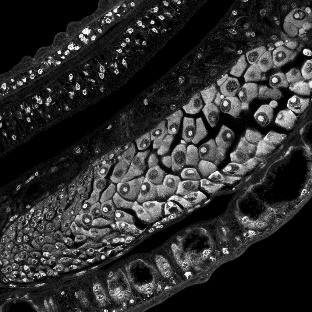

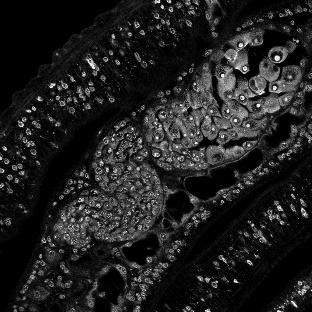

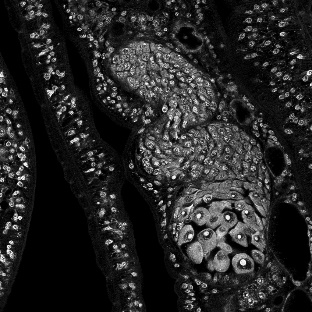


Suppl. Fig. S6: **A**, following RNAi, transcript levels in males were reduced down to 50% - 10% as determined by qRT-PCR using Sm*letm1* (Smp_065110) as a proven reference for gene expression analysis under *in vitro*-culture conditions (Haeberlein et al. 2019) (n = 3). **B**, summary of re-pairing frequencies of *S. mansoni* males with group 1 females (previous pairing experience) *in vitro* following RNAi with dsRNA targeting Sm*tdc-1* (Smp_135230) and Sm*ddc-1* (Smp_171580), either individually (grey and orange lines) or in combination (yellow line; n = 2; each n with 10 worms of both genders). Control, untreated worms maintained *in vitro* for re-pairing under the same conditions (blue line; n = 3; each n with 10 worms of both genders). No statistically significant differences were observed. **C**, summary of egg counts over the observation period of 21 d. Significantly lower numbers of eggs occurred in the treatment groups compared to the control (n = 3). **A** and **C,** statistics were done by one-way ANOVA (GraphPad Prism 7). Data show the mean ± SEM of three separate experiments, and statistically significant differences are shown as *p<0.05, **p<0.01, and ***p<0.001. **D**, CLSM analyses showed lower numbers of differentiated oocytes in both treatment groups (Sm*tdc-1 and* Sm*ddc-1*) compared to the respective control at day 21 after re-pairing. f = female, m = male, mo = mature oocytes, io, immature oocytes; scale bars 50 µm.

**Supplementary Figure 7:**

**RNAi against Sm*tdc-1* and Sm*ddc-1* had no significant influence on the morphology of males**

**
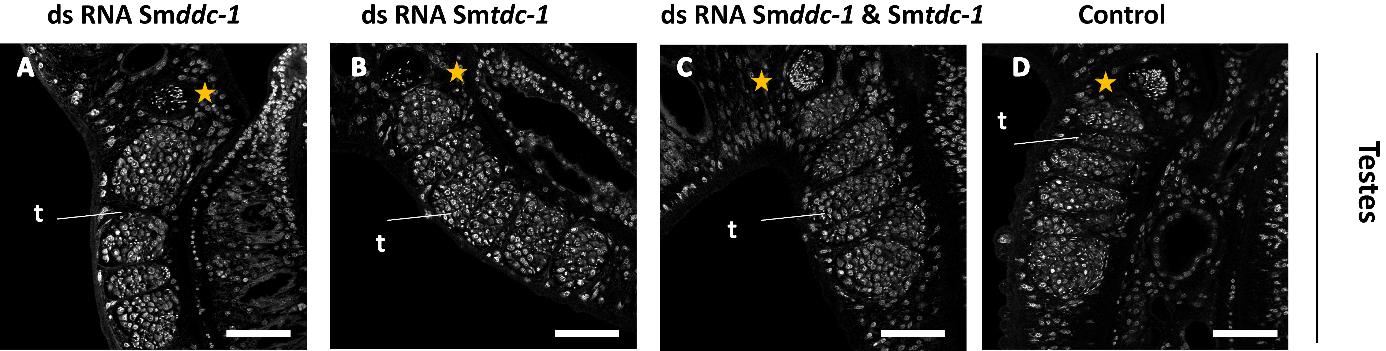
**

Suppl. Fig. S7: By CLSM, we analysed the morphology of the male partners of couples treated with dsRNA against Sm*ddc-1* (**A**), Sm*tdc-1* (**B**), or the combination of both (**C**). Compared to untreated males (D, control), those of the treatment groups showed no obvious difference. In all cases, testes size, occurrence of spermatogonia, and differentiated sperm appeared normal. *, sperm vesicle; scale bars: 50 µm.

**Supplementary tables**

**Supplementary Table 1:**

**Primes used in this study**

| Primer name | Application | Sequence (5’-3’) |
| --- | --- | --- |
| Smp_171580_ pJC 53.2_s | Cloning | ATCCAGCTTGCACAGAACTTGA |
| Smp_171580_ pJC 53.2_as | Cloning | AGTTCCCAAAGTTGCACAGC |
| Smp_171580_ qPCR_For | qRT-PCR | AGTCGCTCTATTGGCTGCAC |
| Smp_171580_qPCR_Rev | qRT-PCR | CTGAACTATGTGCCTGATCCGA |
| Smp_135230_ pJC 53.2_s | Cloning | AGCGGAATTGGTGGAGGTGTA |
| Smp_135230_ pJC 53.2_as | Cloning | GCAGCGTCCACATGTAACCA |
| Smp_135230_ qPCR_For | qRT-PCR | TGGGTTTTCATGGGTTGCAAG |
| Smp_135230_ qPCR_Rev | qRT-PCR | TACACCTCCACCAATTCCGC |
| Smp_065110_qPCR_For | qRT-PCR | CGTGGAATGCGTTCAGTTGG |
| Smp_065110_qPCR_ Rev | qRT-PCR | GAAGCTGATGGAGGTAATTGAG |
| Smp_335630_pJC 53.2_s | Cloning | CTCTTGGTTGTGGGTATAAG |
| Smp_335630_pJC 53.2_as | Cloning | CATGTTCGTCATTACGGTAC |
| pJC 53.2_sequencing | Cloning | TTCTGCGGACTGGCTTTCTAC |
| pJC 53.2_T7_extended primer | dsRNA and WISH  (riboprobes) | CCTAATACGACTCACTATAGGGAG |
